# Global, regional, and national burden of mortality associated with short-term temperature variability from 2000–19: a three-stage modelling study

**DOI:** 10.1016/S2542-5196(22)00073-0

**Published:** 2022-05

**Authors:** Yao Wu, Shanshan Li, Qi Zhao, Bo Wen, Antonio Gasparrini, Shilu Tong, Ala Overcenco, Aleš Urban, Alexandra Schneider, Alireza Entezari, Ana Maria Vicedo-Cabrera, Antonella Zanobetti, Antonis Analitis, Ariana Zeka, Aurelio Tobias, Baltazar Nunes, Barrak Alahmad, Ben Armstrong, Bertil Forsberg, Shih-Chun Pan, Carmen Íñiguez, Caroline Ameling, César De la Cruz Valencia, Christofer Åström, Danny Houthuijs, Do Van Dung, Dominic Royé, Ene Indermitte, Eric Lavigne, Fatemeh Mayvaneh, Fiorella Acquaotta, Francesca de’Donato, Shilpa Rao, Francesco Sera, Gabriel Carrasco-Escobar, Haidong Kan, Hans Orru, Ho Kim, Iulian-Horia Holobaca, Jan Kyselý, Joana Madureira, Joel Schwartz, Jouni J K Jaakkola, Klea Katsouyanni, Magali Hurtado Diaz, Martina S Ragettli, Masahiro Hashizume, Mathilde Pascal, Micheline de Sousa Zanotti Stagliorio Coélho, Nicolás Valdés Ortega, Niilo Ryti, Noah Scovronick, Paola Michelozzi, Patricia Matus Correa, Patrick Goodman, Paulo Hilario Nascimento Saldiva, Rosana Abrutzky, Samuel Osorio, Tran Ngoc Dang, Valentina Colistro, Veronika Huber, Whanhee Lee, Xerxes Seposo, Yasushi Honda, Yue Leon Guo, Michelle L Bell, Yuming Guo

**Affiliations:** Department of Epidemiology and Preventive Medicine (Y Wu MSc, S Li PhD, Q Zhao PhD, B Wen MSc, Prof Y Guo PhD) and Climate, Air Quality Research Unit (Y Wu, S Li, B Wen, Prof Y Guo), School of Public Health and Preventive Medicine, Monash University, Melbourne, VIC, Australia; Department of Epidemiology, School of Public Health, Cheeloo College of Medicine, Shandong University, Jinan, China (Q Zhao); Department of Public Health, Environments and Society (Prof A Gasparrini PhD, A M Vicedo-Cabrera PhD, Prof B Armstrong PhD), Centre for Statistical Methodology (Prof A Gasparrini), and Centre on Climate Change & Planetary Health (Prof A Gasparrini), London School of Hygiene & Tropical Medicine, London, UK; Shanghai Children’s Medical Centre, Shanghai Jiao Tong University, Shanghai, China (Prof S Tong PhD); School of Public Health, Institute of Environment and Population Health, Anhui Medical University, Hefei, China (Prof S Tong); Center for Global Health, Nanjing Medical University, Nanjing, China (Prof S Tong); School of Public Health and Social Work, Queensland University of Technology, Brisbane, QLD, Australia (Prof S Tong); National Agency for Public Health of the Ministry of Health, Labour and Social Protection of the Republic of Moldova, Chișinău, Moldova (A Overcenco PhD); Institute of Atmospheric Physics, Czech Academy of Sciences, Prague, Czech Republic (A Urban PhD, J Kyselý PhD); Faculty of Environmental Sciences, Czech University of Life Sciences, Prague, Czech Republic (A Urban, J Kyselý); Institute of Epidemiology, Helmholtz Zentrum München—German Research Center for Environmental Health, Neuherberg, Germany (A Schneider PhD); Faculty of Geography and Environmental Sciences, Hakim Sabzevari University, Sabzevar, Iran (A Entezari PhD, F Mayvaneh MSc); Institute of Social and Preventive Medicine (A M Vicedo-Cabrera) and Oeschger Center for Climate Change Research (A M Vicedo-Cabrera), University of Bern, Bern, Switzerland; Department of Environmental Health, Harvard TH Chan School of Public Health, Harvard University, Boston, MA, USA (A Zanobetti PhD, B Alahmad MPH, Prof J Schwartz PhD); Department of Hygiene, Epidemiology and Medical Statistics, National and Kapodistrian University of Athens, Athens, Greece (A Analitis PhD, Prof K Katsouyanni PhD); Institute for Environment, Health and Societies, Brunel University London, London, UK (A Zeka PhD); Institute of Environmental Assessment and Water Research, Spanish Council for Scientific Research, Barcelona, Spain (A Tobias PhD); School of Tropical Medicine and Global Health, Nagasaki University, Nagasaki, Japan (A Tobias, X Seposo PhD); Department of Epidemiology (B Nunes PhD) and Environmental Health Department (J Madureira PhD), Instituto Nacional de Saúde Dr Ricardo Jorge, Porto, Portugal; Centro de Investigação em Saúde Pública, Escola Nacional de Saúde Pública, Universidade NOVA de Lisboa, Lisbon, Portugal (B Nunes); Department of Public Health and Clinical Medicine, Umeå University, Umeå, Sweden (Prof B Forsberg PhD, C Åström PhD); National Institute of Environmental Health Science, National Health Research Institutes, Zhunan, Taiwan (S-C Pan MSc, Prof Y L Guo PhD); Department of Statistics and Computational Research, Universitat de València, València, Spain (C Íñiguez PhD); CIBER of Epidemiology and Public Health, Madrid, Spain (C Íñiguez, D Royé PhD); National Institute for Public Health and the Environment (RIVM), Centre for Sustainability and Environmental Health, Bilthoven, Netherlands (C Ameling BS, D Houthuijs PhD); Department of Environmental Health, National Institute of Public Health, Cuernavaca Morelos, Mexico (C De la Cruz Valencia MSc, Prof M Hurtado Diaz PhD); Department of Environmental Health, Faculty of Public Health, University of Medicine and Pharmacy at Ho Chi Minh City, Ho Chi Minh City, Vietnam (D V Dung PhD, T N Dang PhD); Department of Geography, University of Santiago de Compostela, Santiago de Compostela, Spain (D Royé); Institute of Family Medicine and Public Health, University of Tartu, Tartu, Estonia (E Indermitte PhD, H Orru PhD); School of Epidemiology & Public Health, Faculty of Medicine, University of Ottawa, Ottawa, ON, Canada (Prof E Lavigne PhD); Air Health Science Division, Health Canada, Ottawa, ON, Canada (Prof E Lavigne); Department of Earth Sciences, University of Torino, Turin, Italy (F Acquaotta PhD); Department of Epidemiology, Lazio Regional Health Service, Rome, Italy (F de’Donato PhD, P Michelozzi MSc); Norwegian Institute of Public Health, Oslo, Norway (S Rao PhD); Department of Statistics, Computer Science and Applications “G Parenti”, University of Florence, Florence, Italy (F Sera MSc); Health Innovation Lab, Institute of Tropical Medicine “Alexander von Humboldt”, Universidad Peruana Cayetano Heredia, Lima, Peru (G Carrasco-Escobar MSc); Scripps Institution of Oceanography, University of California San Diego, La Jolla, CA, USA (G Carrasco-Escobar); Department of Environmental Health, School of Public Health, Fudan University, Shanghai, China (Prof H Kan PhD); Graduate School of Public Health, Seoul National University, Seoul, South Korea (Prof H Kim PhD); Faculty of Geography, Babeş-Bolyai University, Cluj-Napoca, Romania (I-H Holobaca PhD); EPIUnit—Instituto de Saúde Pública, Universidade do Porto, Porto, Portugal (J Madureira); Laboratório para a Investigação Integrativa e Translacional em Saúde Populacional (ITR), Porto, Portugal (J Madureira); Center for Environmental and Respiratory Health Research (CERH), University of Oulu, Oulu, Finland (Prof J J K Jaakkola PhD, N Ryti PhD); Medical Research Center Oulu (MRC Oulu), Oulu University Hospital and University of Oulu, Oulu, Finland (Prof J J K Jaakkola, N Ryti); School of Population Health and Environmental Sciences, King’s College London, London, UK (Prof K Katsouyanni); Swiss Tropical and Public Health Institute, Basel, Switzerland (M S Ragettli PhD); University of Basel, Basel, Switzerland (M S Ragettli); Department of Global Health Policy, Graduate School of Medicine, The University of Tokyo, Tokyo, Japan (Prof M Hashizume PhD); Santé Publique France, Department of Environmental and Occupational Health, French National Public Health Agency, Saint Maurice, France (M Pascal PhD); Department of Pathology, Faculty of Medicine (M de Sousa Zanotti Stagliorio Coélho PhD) and Department of Environmental Health (S Osorio MSc), University of São Paulo, São Paulo, Brazil; Department of Public Health, Universidad de los Andes, Santiago, Chile (N V Ortega MSc, P M Correa MSc); Gangarosa Department of Environmental Health, Rollins School of Public Health, Emory University, Atlanta, GA, USA (N Scovronick PhD); School of Physics, Technological University Dublin, Dublin, Ireland (Prof P Goodman PhD); INSPER, São Paulo, Brazil (Prof P H Nascimento Saldiva PhD); Universidad de Buenos Aires, Facultad de Ciencias Sociales, Instituto de Investigaciones Gino Germani, Buenos Aires, Argentina (R Abrutzky MSc); Department of Quantitative Methods, School of Medicine, University of the Republic, Montevideo, Uruguay (V Colistro MSc); IBE-Chair of Epidemiology, LMU Munich, Munich, Germany (V Huber PhD); Department of Physical, Chemical and Natural Systems, Universidad Pablo de Olavide, Sevilla, Spain (V Huber); School of the Environment, Yale University, New Haven, CT, USA (W Lee PhD, Prof M L Bell PhD); Department of Occupational and Environmental Medicine, School of Medicine, Ewha Womans University, Seoul, South Korea (W Lee); Center for Climate Change Adaptation, National Institute for Environmental Studies, Tsukuba, Japan (Prof Y Honda PhD); Environmental and Occupational Medicine, National Taiwan University College of Medicine and NTU Hospital (Prof Y L Guo) and Graduate Institute of Environmental and Occupational Health Sciences, National Taiwan University College of Public Health (Prof Y L Guo), National Taiwan University, Taipei, Taiwan

## Abstract

**Background:**

Increased mortality risk is associated with short-term temperature variability. However, to our knowledge, there has been no comprehensive assessment of the temperature variability-related mortality burden worldwide. In this study, using data from the MCC Collaborative Research Network, we first explored the association between temperature variability and mortality across 43 countries or regions. Then, to provide a more comprehensive picture of the global burden of mortality associated with temperature variability, global gridded temperature data with a resolution of 0·5° × 0·5° were used to assess the temperature variability-related mortality burden at the global, regional, and national levels. Furthermore, temporal trends in temperature variability-related mortality burden were also explored from 2000–19.

**Methods:**

In this modelling study, we applied a three-stage meta-analytical approach to assess the global temperature variability-related mortality burden at a spatial resolution of 0·5° × 0·5° from 2000–19. Temperature variability was calculated as the SD of the average of the same and previous days’ minimum and maximum temperatures. We first obtained location-specific temperature variability related-mortality associations based on a daily time series of 750 locations from the Multi-country Multi-city Collaborative Research Network. We subsequently constructed a multivariable meta-regression model with five predictors to estimate grid-specific temperature variability related-mortality associations across the globe. Finally, percentage excess in mortality and excess mortality rate were calculated to quantify the temperature variability-related mortality burden and to further explore its temporal trend over two decades.

**Findings:**

An increasing trend in temperature variability was identified at the global level from 2000 to 2019. Globally, 1 753 392 deaths (95% CI 1 159 901–2 357 718) were associated with temperature variability per year, accounting for 3·4% (2·2–4·6) of all deaths. Most of Asia, Australia, and New Zealand were observed to have a higher percentage excess in mortality than the global mean. Globally, the percentage excess in mortality increased by about 4·6% (3·7–5·3) per decade. The largest increase occurred in Australia and New Zealand (7·3%, 95% CI 4·3–10·4), followed by Europe (4·4%, 2·2–5·6) and Africa (3·3, 1·9–4·6).

**Interpretation:**

Globally, a substantial mortality burden was associated with temperature variability, showing geographical heterogeneity and a slightly increasing temporal trend. Our findings could assist in raising public awareness and improving the understanding of the health impacts of temperature variability.

**Funding:**

Australian Research Council, Australian National Health & Medical Research Council.

## Editorial note

the *Lancet* Group takes a neutral position with respect to territorial claims in published maps and institutional affiliations.

## Introduction

Climate change is a major public health concern in the 21st century. Climate change affects both the global mean surface temperature and its variability, resulting in more frequent extreme weather events and unstable weather.^1–3^ Globally, non-optimum temperatures have been identified as an important indicator of climate change given the largely recognised warming trend, and as one of the leading causes of the global burden of diseases.^4–6^ However, temperature variability, another challenging aspect of climate change that reflects weather stability, has less public awareness and has been less investigated compared with non-optimum temperatures.^7,8^

Temperature variability can be measured in many ways, such as diurnal temperature range (DTR), reflecting intra-day temperature variability, and temperature change between two adjacent days (TCN), reflecting inter-day temperature variability.^9,10^ Both DTR and TCN are associated with various health outcomes. Given that unstable weather is a continuous process that takes time for human beings to adapt to, a composite index of temperature variability has been developed using the SD of daily minimum and maximum temperatures (T_min_ and T_max_) during several exposure days, to assess the combined effect of both intra-day and inter-day temperature variability and their lagged effects.^11,12^

Some studies have reported adverse health impacts of short-term exposure to temperature variability, showing a significant association between temperature variability and mortality risk.^11–14^ Our previous study based on Multi-Country Multi-City (MCC) Collaborative Research Network data showed significant but varied associations between temperature variability and mortality risk across 12 countries with various climate patterns, indicating that temperate variability can affect the entire population, but poses a higher risk to particular population groups (eg, children, older people, and people with pre-existing illness).^11,12,15^ Although several studies have investigated the association between temperature variability and mortality,^11–14^ few have assessed the absolute mortality burden associated with temperature variability.

We have seen an increased mortality burden attributable to hot temperatures,^16^ and it would be beneficial to explore how temperature variability-related mortality burden changes over time. Since the pre-industrial era, the global temperature has increased by more than 1°C.^17^ However, temperature variability was observed to vary in time and space without consistent temporal patterns.^18–20^ The reasons for this temporal–spatial variation can be multifaceted. Dynamic temperature changes are highly correlated with long-wave radiation fluxes, which depend on both natural (eg, atmospheric circulation, cloud cover, and precipitation) and anthropogenic factors (eg, over-exploitation and excessive grazing) that vary from region to region.^21–24^ Therefore, it is necessary to understand the temporal trend in temperature variability-related mortality burden across the globe and to be able to make comparisons between regions during the same time window.

In this study, using data from the MCC Collaborative Research Network, we first explored the association between temperature variability and mortality across 43 countries or regions. Then, to provide a more comprehensive picture of the global burden of mortality associated with temperature variability, global gridded temperature data with a resolution of 0·5° × 0·5° were used to assess the temperature variability-related mortality burden at the global, regional, and national levels. Furthermore, temporal trends in temperature variability-related mortality burden were also explored from 2000–19.

## Methods

### Data sources

Daily death counts extracted from the MCC Collaborative Research Network database were used in this study. 750 cities across 43 countries or regions were included. International Classification of Diseases, 9th and 10th revision (ICD-9 and ICD-10) codes were used to identify causes of death. We extracted the data series on non-external causes of death (ICD-9: 0–799; ICD-10: A00–R99) or, if not available, all-cause mortality. Descriptive statistics by countries or regions are shown in the appendix (pp 3–4). Only 0·09% of all-cause death data were missing (appendix pp 5–6).

Daily 1-h T_max_ and T_min_ data at 0·5° × 0·5° latitude–longitude resolution during 1979–2019 were collected from the Climate Prediction Centre Global Temperature data provided by the National Oceanic and Atmospheric Administration Physical Sciences Laboratory. The dataset originated from T_min_ and T_max_ data from 6000–7000 stations across the globe and is interpolated using the Shepard algorithm with orographic consideration to develop gridded data.^25^ The daily mean temperature was found by calculating the mean T_min_ and T_max_. Temperature variability was calculated as the SD of the daily T_min_ and T_max_ for the current day (lag0) and lag days (eg, lag1, lag2, … lag7).^11^ For example, temperature variability over lag 0–3 days was calculated as follows:

$$\text{SD}\;(T_{max-lag0},\;T_{min-lag0},\;T_{max-lag1},\;T_{min-lag1},\;T_{max-lag2},\;T_{min-lag2},\;T_{max-lag3},\;T_{min-lag3})$$

Data on the global gross domestic product (GDP) and population in 0·5° grid between 1980 and 2020 by 10 years were obtained from the Global Carbon Project.^26^ GDP and population data were linearly interpolated over time to generate values for each year. GDP per capita was calculated by dividing the GDP by the population. All GDP per capita data were adjusted to 2010 $US.

We obtained country-specific mortality rates for each year from the World Bank. For each year, the mean daily deaths for each grid cell were computed as the product of the grid-specific population and annual mortality rate of the country where the grid cell was located, divided by the number of days in a year. Mortality rates were assumed to be identical across all grid cells in the same country, which is widely used in the Global Burden of Diseases, Injuries, and Risk Factors Study.^27–30^

Ethics approval was not required for our analysis of aggregate anonymised data from the MCC Collaborative Research Network database.

### Statistical analysis

Using a seasonal-trend decomposition procedure based on locally weighted smoothing (STL), we decomposed time-series data for temperature variability into seasonal, trend, and remainder components.^31^ We applied the STL method to each grid cell to decompose the time-series data of temperature variability and extract the long-term trend. The global trend of temperature variability was then obtained by calculating the mean long-term trends across all grid cells.

A three-stage approach established in previous research was applied to quantify the global temperature variability-related mortality burden at a spatial resolution of 0·5° × 0·5°.^16,32^ Briefly, we firstly obtained location-specific temperature variability–mortality associations based a on daily time series of 750 locations from the MCC Collaborative Research Network. Then, a multivariable meta-regression model was built with five predictors to estimate grid-specific temperature variability-related mortality associations across the globe. Finally, percentage excess in mortality and excess mortality rate were calculated to quantify the temperature variability-related mortality burden and to further explore its temporal trend over two decades.

In the first stage, a generalised linear regression model with a quasi-Poisson family was applied in each location to obtain location-specific effect estimates for temperature variability–mortality association. The equation was as follows:^33^

$$Y_{it}\sim \text{Poisson}(\mu ;\theta )\;\text{E}\left(Y_{it}\right)=\text{exp}(\alpha_{\text{i}}+\beta_{\text{i}}TV_{\text{it}}+\text{cb}\left(Temp_{it},lag=21\right)+\text{ns}(Time_{\text{it}},\text{df}=7/\text{year})+\gamma_{\text{i}}DOW_{it})\;VAR\left(Y_{it}\right)=\theta \mu$$

where *Y*_*it*_ denotes daily death count in location *i* on day *t*; α_*i*_ represents the intercept in location *i*; β_*i*_ and γ_*I*_ represent the coefficients in location *i*; and *TV*_*it*_ stands for the linear function of temperature variability.^11^ c*b*(*Temp*_*it*_,lag = 21), built by distributed-lag nonlinear models (DLNMs), is a two-dimensional, parameterised cross-basis function of daily mean temperature. One dimension is for the space of temperature, featuring the non-linear effect of temperature, with a natural cubic spline function with three internal knots placed at the 25th, 50th, and 75th percentile of the location-specific temperature distribution. The other dimension is for the space of lag, featuring the delayed effect of temperature over 21 days of lag, with a natural cubic spline function with two internal knots placed at equally spaced values in the log scale, plus intercept; n*s*(*Time*_*it*_,*df = 7/year*) is a natural cubic spline for time with seven degrees of freedom per year. The number of degrees of freedom determines the flexibility of the spline function. We chose seven degrees of freedom per year to provide adequate control for long-term trend and seasonality. *DOW*_*it*_ stands for the day of the week coded as a categorical variable. *VAR*(*Y*_*it*_) and *μ* denote the variance and expectation of *Y*_*it*_ and θ is an overdispersion parameter. The association between temperature variability and mortality was presented as the relative risk (RR) with 95% CI associated with each 1°C increase in temperature variability. The percentage change in mortality with an IQR increase in temperature variability was also computed.

In the second stage, a multivariable meta-regression model was built to quantify the relationship between the location-specific effect estimates obtained from the first stage and a set of independent location-specific explanatory variables from each location. We identified five explanatory variables that were documented in previous studies to contribute to the heterogeneity of location-specific effect estimates, including continents, five climate groups of Köppen climate classification, GDP per capita, the yearly average of daily mean temperature, and the range of daily mean temperature.^16,34^ All five variables should have global data at the grid cell level. Mid-year GDP per capita (the middle year of the study period for each location) was calculated to reflect the mean GDP per capita for each location. The performance of multivariable meta-regression models was checked by the *I*^2^ statistic. The final model showed an *I*^2^ of 22·67% (appendix p 7). The coefficients of five explanatory variables were extracted from the constructed model and used in the third stage to estimate the temperature variability–mortality association at the grid cell level.

In the third stage, the fitted meta-regression model obtained in the second stage with five grid-specific explanatory variables was used to estimate the temperature variability–mortality association between 2000 and 2019 at the grid cell level.

We calculated the daily excess deaths associated with temperature variability in each grid cell using the following equation:

$$RR_{it}=\text{exp}\left(\beta_{per\;1\;{}^{\circ}\text{C }\text{increase}}\times TV_{it}\right)$$

$$ED_{it}=\left(RR_{it-1}\right)\times D_{i}$$

where *RR*_*it*_ is the RR of grid cell *i* on day; β_*per 1°C increase*_ is the grid-specific association; *TV*_*it*_ is the temperature variability of grid cell *i* on day *t*; *ED*_*it*_ stands for the excess deaths in grid cell *i* on day *t*; and *D*_*i*_ is the daily deaths in each grid cell.

The total number of excess deaths was computed as the sum of daily excess deaths for each year and the entire study period at the global, regional, and national levels. The percentage excess in mortality was calculated by the ratio of excess deaths to total deaths. The average annual percentage excess over 20 years was further computed. Annual excess deaths per 100 000 residents (excess death rate) were also presented. For each region or continent, we calculated the percentage change per decade in both percentage excess in mortality and excess death rate, using a linear regression model considering a Gaussian distribution of percentage excess and excess death rate on the log scale. The 95% CI of percentage change per decade was obtained based on 1000 bootstrap replicates. To make our results easier to follow, we applied the length of exposure of 8 days (temperature variability 0–7) in the main analyses. Results for other lengths of exposure (from temperature variability 0–1 to temperature variability 0–6 and from temperature variability 0–8 to temperature variability 0–10) were shown in the sensitivity analyses.

Several other sensitivity analyses were done to test the robustness of our results, as follows: extending the maximum lag periods of mean temperature from 21 days to 24 days and 28 days; using alternative degrees of freedom values for time trend (from seven degrees of freedom per year to six degrees of freedom and eight degrees of freedom per year) and lag days of mean temperature (from four degrees of freedom to five degrees of freedom and six degrees of freedom); and controlling the potential effect of relative humidity using a natural cubic spline with three degrees of freedom. A detailed description of sensitivity analyses is shown in the appendix (p 2). The significance of the difference in results between primary analyses and sensitivity analyses was tested using a fixed-effect meta-regression model. Additionally, as we used the counter-factual scenario of no variation in the main analyses, excess deaths represent those that would not have occurred if temperature variability never exceeded 0°C. Considering that temperature variability is less likely to be 0, we also calculated the excess deaths under the counterfactual scenario of the grid-specific minimum temperature variability, by excluding the excess deaths associated with temperature variability ranging from 0 to minimum value, to assess the mortality burden in the more stringent criteria.

R (version 3.6.2) was used for all analyses. The R packages dlnm (version 2.4.2), mixmeta (version 1.0.8), and stR were used to perform DLNMs, meta-regression models, and seasonal-trend decomposition, respectively.

### Role of the funding source

The funders of the study had no role in study design, data collection, data analysis, data interpretation, or writing of the report.

## Results

**Figure 1:**
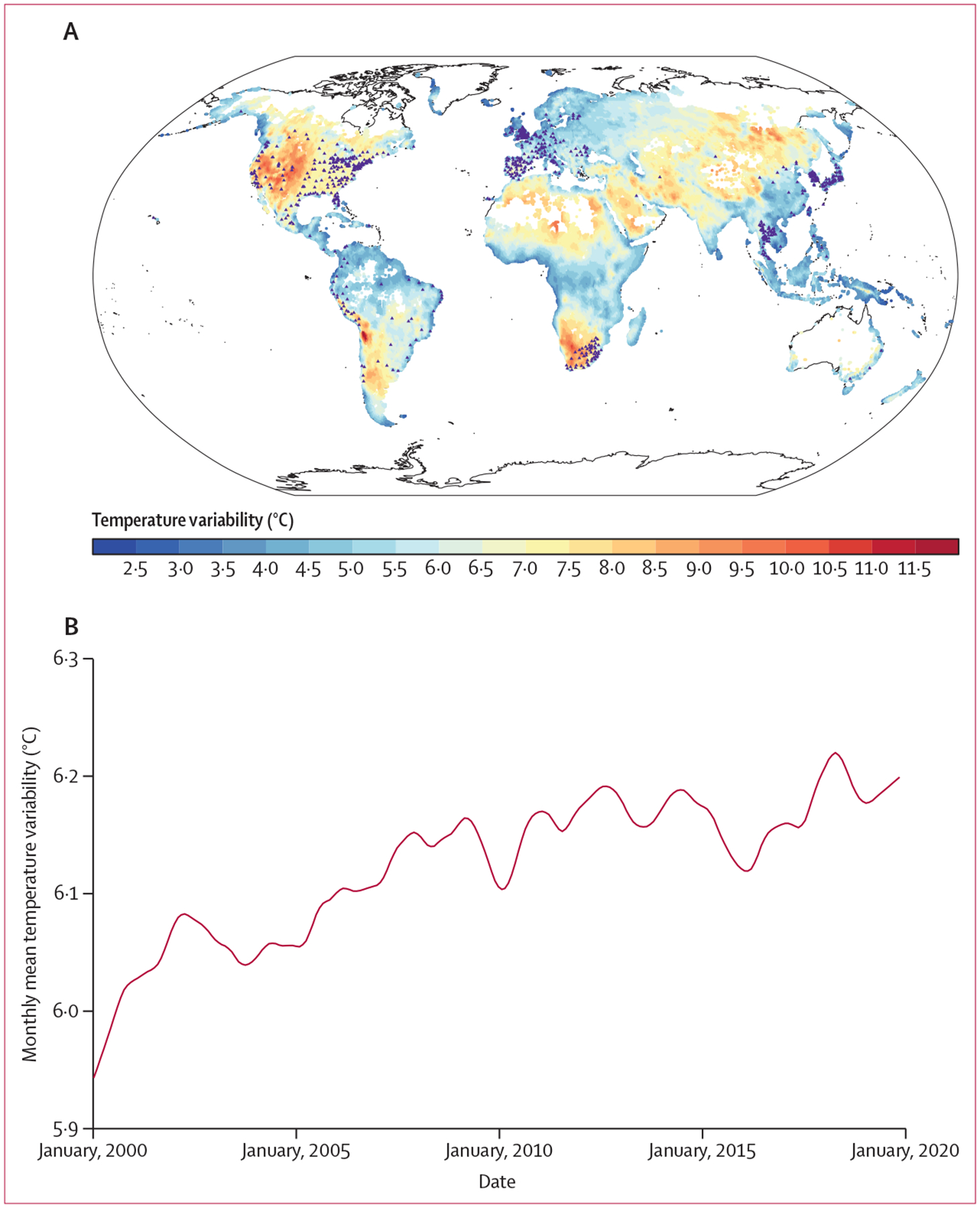
Mean annual temperature variability at a spatial resolution of 0·5° × 0·5° (A) and the long-term trend in annual temperature variability after seasonal-trend decomposition (B) across the globe from 2000–19 Triangles in A represent the 750 locations used in the first-stage analysis.

The mean annual temperature variability between 2000 and 2019 is shown in figure 1A. Globally, a large variation in temperature variability was observed. Several regions were identified to have higher temperature variability, such as North America, southern Africa, and northern Africa. The global mean temperature variability was 6·0°C (SD 1·3) in 2000 and 6·2°C (1·3) in 2019 (appendix p 8). After seasonal-trend decomposition, a rising long-term trend in temperature variability was found across the globe (figure 1B; appendix p 9). Among all regions, Australia and New Zealand had the largest increase in annual temperature variability (appendix p 9).

**Figure 2:**
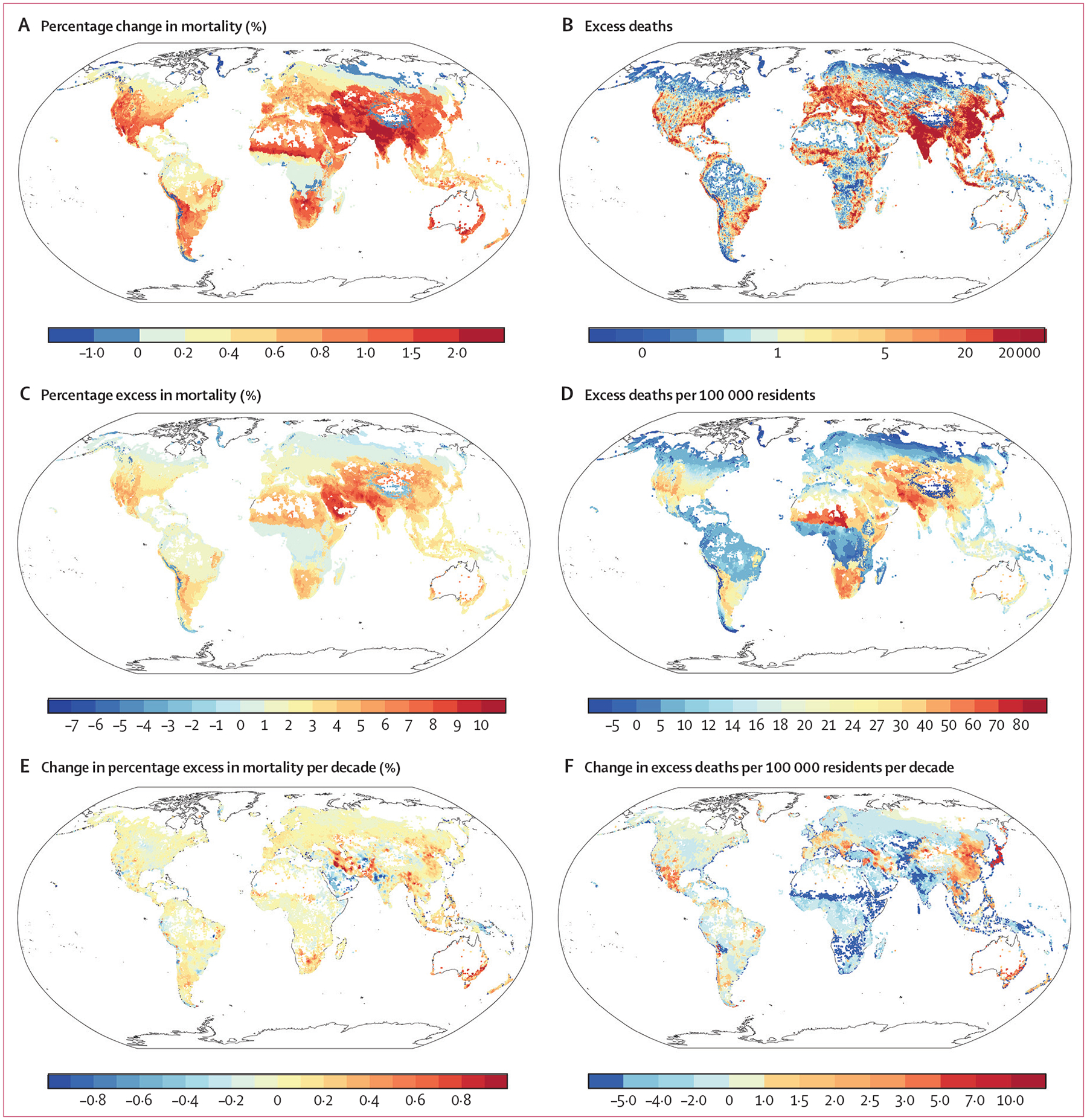
Percentage change in mortality associated with an IQR (for each grid cell) increase in temperature variability (A), mean annual excess deaths (B), percentage excess in mortality (C), excess deaths per 100 000 residents (D), change in percentage excess in mortality per decade (E), and change in excess deaths per 100 000 residents per decade (F) due to temperature variability in 2000–19 at a spatial resolution of 0·5° × 0·5° The scale in E represents change in percentage points, not percentage change.

In general, each IQR increase in temperature variability was associated with a mean 0·7% increase in mortality across all grid cells, with a median value of 0·6% (IQR 0·3–1·0; figure 2A). The country-specific temperature variability related-mortality risks are shown in the appendix (pp 10–14). The geographical variation was observed globally. South Asia had the highest mortality risk associated with temperature variability (figure 2A). Hotspot areas with the biggest contribution to excess deaths were recognised in most parts of south and east Asia (figure 2B). A higher percentage excess in mortality was observed in most of west Asia, the south of middle Asia, and the north of south Asia (figure 2C). The junction of western Africa and central Africa had the highest excess death rate (per 100 000 residents; figure 2D). The changing nature per decade of the percentage excess is shown in figure 2E. The percentage excess on the southeast coast of Australia increased markedly, along with separate areas in the north of western Asia. The excess death rates (per 100 000 residents) were shown to increase in east Asia, the south of North America, and the southeast coast of Australia (figure 2F).

**Figure 3:**
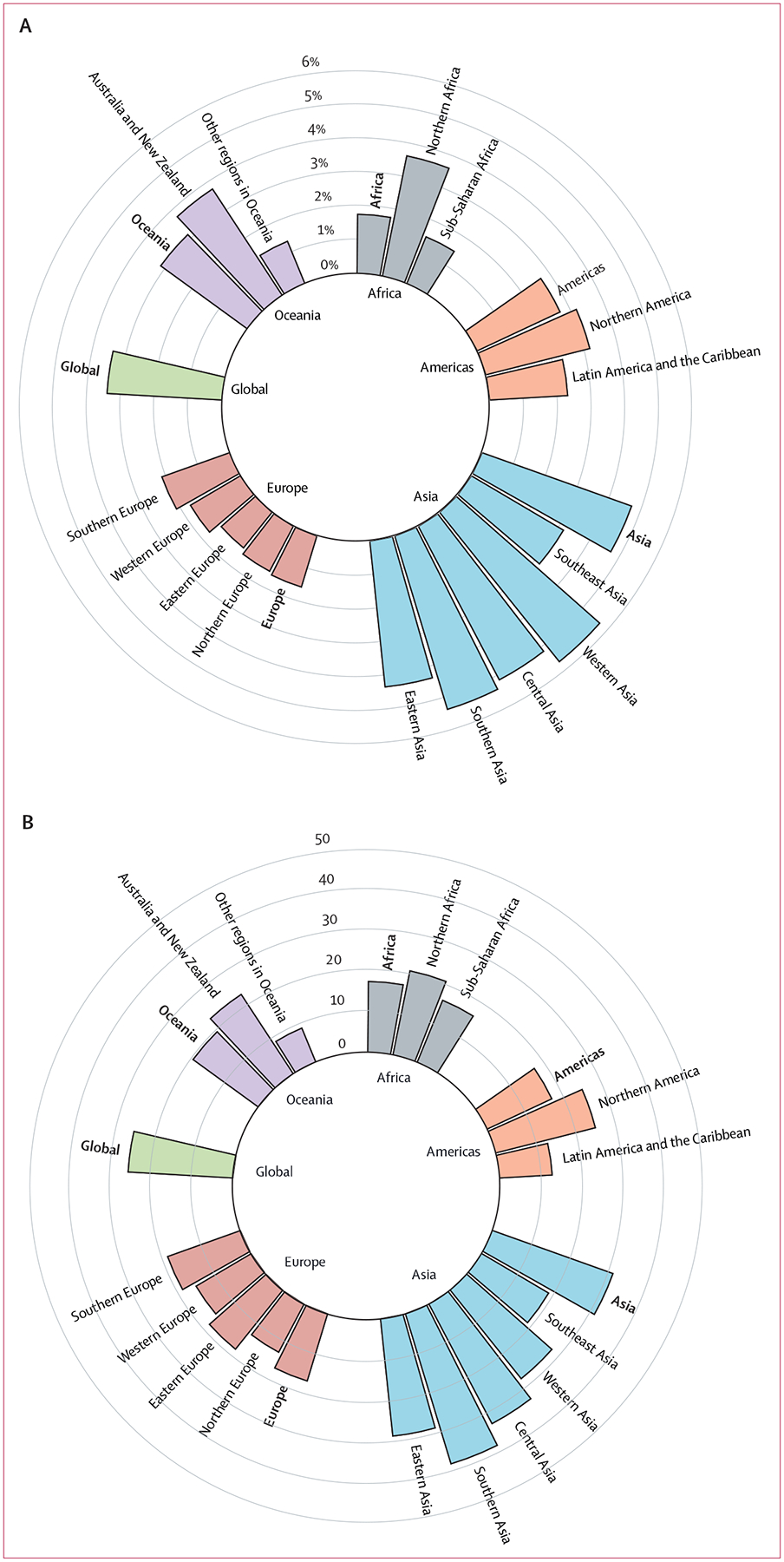
Average annual percentage excess in mortality (A) and excess deaths per 100 000 residents (B) due to temperature variability in 2000–19 by continent and region

From 2000–19, globally, 1 753 392 (95% CI 1 159 901 to 2 357 718) excess deaths was associated with temperature variability per year (table), accounting for 3·4% (95% CI 2·2 to 4·6) of the total deaths and 26 (17 to 35) excess deaths per 100 000 residents (figure 3; appendix p 15). The three leading continents in terms of percentage excess in mortality were Asia (4·7%), Oceania (3·2%), and the Americas (2·7%; figure 3A; appendix p 15). Southern Asia had the highest excess death rate (39 per 100 000 residents, 95% CI 29 to 48) among all regions, whereas the lowest value was observed for other regions in Oceania (nine per 100 000 residents, −4 to 22; figure 3B; appendix p 15). In addition to the region, climate zones contributed to the variation in excess mortality (appendix p 16). Dry climates had the highest percentage excess in mortality (6·0, 95% CI 4·6 to 7·5).

The global percentage excess in mortality increased from 3·2% (95% CI 2·1 to 4·3) to 3·5% (2·3 to 4·7) between 2000 and 2019, representing an increased rate of 4·6% (3·7 to 5·3) per decade (table). Australia and New Zealand generated the largest increase in percentage excess, increasing from 3·4% (1·3 to 5·4) in 2000 to 4·3% (1·7 to 6·9) in 2019, representing an increased rate of 7·3% (4·3 to 10·4) per decade. The largest decline occurred in other regions in Oceania, with a decreased rate of 5·8% per decade (−18·4 to 5·6; table). Corresponding scatter plots from 2000 to 2019 are shown in the appendix (pp 17–20).

**Figure 4:**
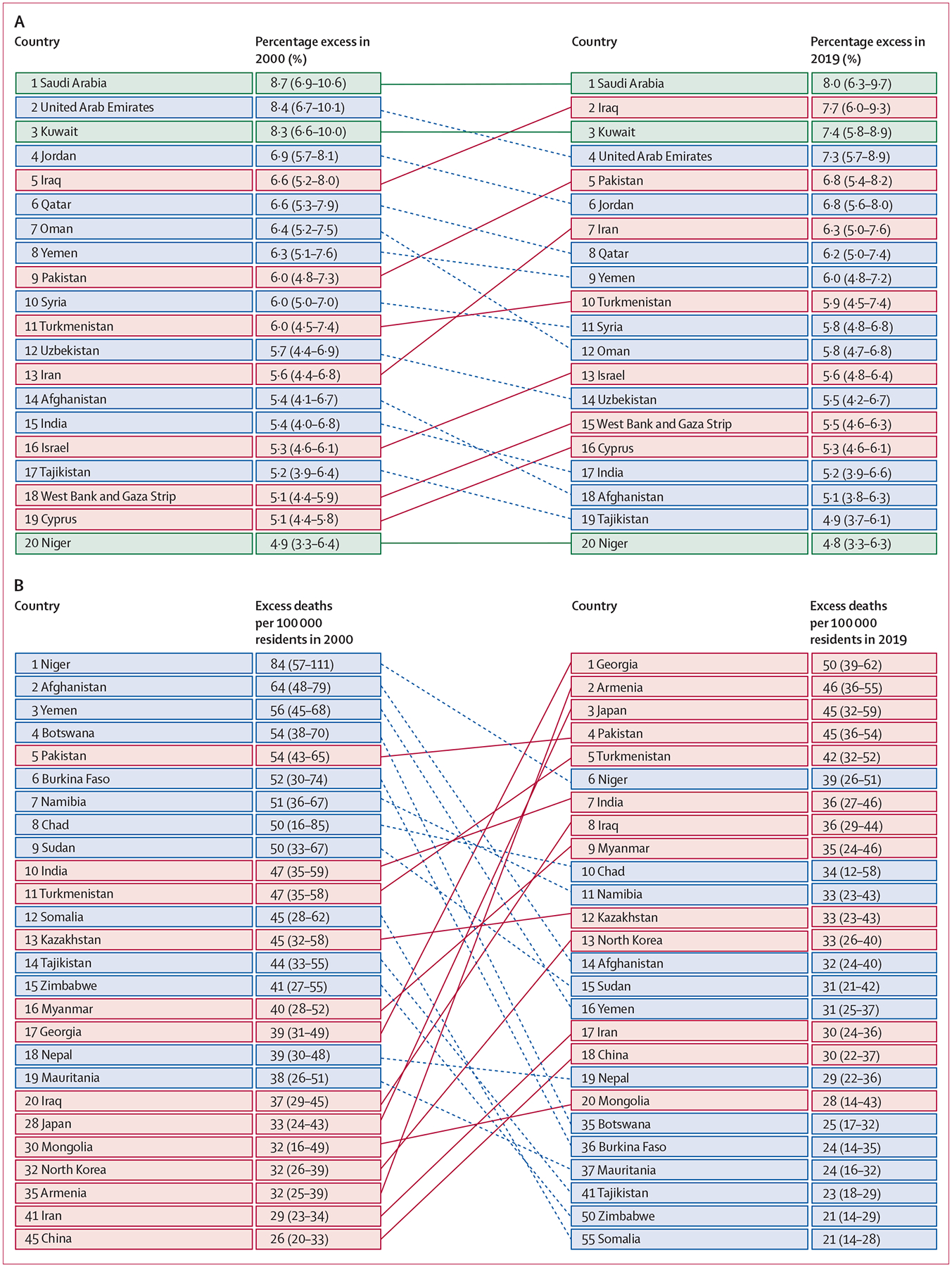
Leading 20 countries for percentage excess in mortality (A) and excess deaths per 100 000 residents (B) in 2000 and 2019

Lists of the top 20 countries ranked by temperature variability-related mortality burden in both 2000 and 2019 included many of the same countries, but the order changed (appendix p 21; figure 4). Among the top ten countries in percentage excess in 2019, four were listed in the current world bank high-income economies, including Saudi Arabia (first), Kuwait (third), United Arab Emirates (fourth), and Qatar (eighth) (figure 4A). Compared with the percentage excess, excess death rates (per 100 000 residents) decreased markedly during the 20 years studied (figure 4B).

In the sensitivity analyses, the mortality burden associated with temperature variability decreased with shorter exposure to temperature variability (appendix p 22). After changing the model parameters, our results changed slightly (appendix pp 23–24). When the counter-factual scenario of grid-specific minimum temperature variability was applied, the percentage excess was 2·2% (95% CI 1·5–2·9), nearly two-thirds of that under the counterfactual scenario of zero temperature variability (appendix p 25).

## Discussion

To our knowledge, this is the largest and first study to use global gridded observation data at a spatial resolution of 0·5° × 0·5° to systematically estimate the global burden of mortality associated with temperature variability and explore its temporal trend over 20 years. From 2000 to 2019, the daily mean value of temperature variability generally increased. A considerable number of deaths were associated with temperature variability per year, causing a substantial mortality burden worldwide. An increasing trend of the percentage excess in mortality was observed during the 20 years studied.

Consistent with previous studies,^13,14,35–37^ we observed an increased mortality risk associated with temperature variability, accounting for a substantial mortality burden. The percentage change in mortality associated with an IQR increase in temperature variability ranged from 0 to 2% for most grid cells, which is similar to previous results based on 12 countries.^11^ The physiological mechanisms underlying this association might relate to thermal adjustment to temperatures through physiological and behavioural responses that are impeded by unstable weather over a short period of time.^8,38^ During these processes, multiple organs can be involved (eg, respiratory, circulatory, and immune systems) by affecting heart rate, blood viscosity, fibrinogen, platelet count, arterial blood pressure, and oxygen uptake.^39–41^ Although the biological mechanisms have not been fully elucidated, they imply a difficult process of thermal adjustment to temperature variability.

To protect human health against temperature variability, proactive countermeasures such as warning systems, community-level responses, and instructions for self-protection are necessary. Many policies have been developed to cope with the threat of climate-related extreme events, for example, warning systems for heatwaves and air pollution.^42,43^ However, policies and strategies rarely exist to effectively cope with the adverse health impacts of temperature variability. Previous investigations reported an estimate of 7·6% for attributable mortality caused by ambient air pollution.^44^ Investigators who separated the hot and cold impact from non-optimum temperatures suggested an excess death ratios of 8·5% for cold-related temperatures and 0·9% for hot-related temperatures.^16^ As suggested by our findings, temperature variability has similar impacts to air pollution and non-optimum temperatures on global mortality. More attention should be paid to the health impacts of temperature variability. One solution is to develop early warning systems of temperature variability, as personal protection behaviours are highly correlated with risk perception. For example, outdoor workers can prepare enough clothes in advance with a timely warning to protect them from sudden temperature changes. Development of guidance on self-protection (eg, stay indoors, take clothes, and take care of vulnerable populations, such as children and older people) with community social programmes will be of great benefit to help people understand what they need to do when temperature fluctuates over a short time period. In the long run, measures to reduce the impact of climate change (eg, clean energy and greenhouse gas emission reduction) should be promoted to fundamentally mitigate global warming, as well as the increasing trend of temperature variability, although these measures might take time to implement and have an impact.^45^ Regions with a higher percentage excess in mortality due to temperature variability (eg, Asia, Australia and New Zealand, and northern Africa) are of great importance to contribute to coordinated actions for health. Some countries, especially developing countries, will suffer disproportionately more from the adverse effects of global climate change, which could be a potential driver for international inequality.^46^

In this study, we observed a small but significant increasing trend in both temperature variability and temperature-related percentage excess in mortality globally. The reduction in the excess death rate from 2000 to 2019 might be largely due to a decrease in mortality rate, whereas almost all regions showed an upward trend in percentage excess in mortality associated with temperature variability, indicating a persistent impact in the past two decades. Although few studies focused on the temporal trends of temperature variability-related mortality burden, investigations can be made through the relevant assessment of similar indicators. For example, a multi-country study of 20 countries or regions projected that a 1·4–10·3% increase in excess deaths attributable to the DTR will happen by the end of this century; the study inferred a more pronounced mortality burden in the future due to more unstable weather than in the past, although there might be an adaptation to climate change benefiting from socioeconomic development and investment in public health.^23,47,48^ More targeted policies should be implemented to avoid the negative health impacts of temperature variability, especially for regions with a higher increasing rate of temperature variability-related mortality burden (eg, Australia and New Zealand, northern Europe, Latin America and the Caribbean, and western Asia).

This study has several strengths. First, to our knowledge, this is the first and largest study to systematically explore the mortality burden associated with temperature variability on a global scale. Compared with previous studies that were restricted to single or several countries,^14,37,49^ this study offers a finer spatial view of the mortality burden associated with temperature variability, which can provide new clues on geographical variations and allow within-country comparisons. Second, this study benefits from global gridded population and climate data. To minimise potential exposure misclassification from aggregating individual exposure to location or country level (aggregation bias), we used exposure data in a 0·5° grid to produce better countrywide and global estimates. Finally, we considered spatiotemporal trends over a 20-year period of fast climate change. The findings of this study provide a better understanding of how temperature variability has affected human health amid inevitable warming trends and gradual acclimatisation to climate change.

This study also has some limitations. We used country-specific mortality rates rather than grid-specific mortality rates because of insufficient data. The assumption of an identical mortality rate across grid cells within the same country is widely used in the Global Burden of Diseases, Injuries, and Risk Factors Study,^27–30^ and should not have a substantial effect on our estimations at country, region, and global levels. However, this fact limits our ability to identify variation in temperature variation-related mortality burden within countries and these limitations should be supplemented in future studies by collection of mortality data at a finer level. Grid cell-specific data (eg, temperature, population, and GDP) applied in this study also introduced uncertainties, as interpolation and downscaling produced prediction errors.

Although previous studies suggested variation in susceptibility to temperature variability across age, sex, and causes of mortality,^15,50^ our study did not characterise these differences because of a lack of age-specific, sex-specific, and cause-specific mortality data at both the grid cell and country level. Future research could complement the evidence provided in this study if relevant data are available. Several predictors that could explain the heterogeneity in the grid-specific temperature variability related-mortality associations were used to estimate these associations. However, we must acknowledge that there could be unexplained heterogeneity contributed by both the paucity of grid-specific data and unknown factors. Further studies are warranted to provide more precise estimates of this association. Owing to the nature of time-series designs, we could not investigate the causal relationship between temperature variability and mortality. Grid cell-specific estimations were based on the temperature variability related-mortality association, but not causation. Finally, MCC data include only 43 countries or regions and have limited information on countries located in the Sahara desert. This paucity of data might affect the accuracy of effect estimates. Although we used Köppen climate classification as one of the predictors in the model, future studies are needed to further explore the association between temperature variability and mortality in the desert area.

In conclusion, this study highlights the substantial mortality burden associated with temperature variability. This burden had a complex pattern of variation globally and a slightly increasing temporal trend over the past two decades. Considering climate change, our findings could assist in raising public awareness and improving the understanding of the impacts of temperature variability on health.

## Supplementary Material

1

## Figures and Tables

**Table:** Percentage excess in mortality and excess deaths per 100 000 residents in 2000 and 2019 and percentage change per decade over 2000–19 by continent and region

	Mean annual excess deaths	Excess in mortality (%)			Excess deaths, per 100 000 residents		
		2000	2019	Change per decade (%)*	2000	2019	Change per decade (%)*
Global	1 753 392 (1 159 901 to 2 357 718)	3·2 (2·1 to 4·3)	3·5 (2·3 to 4·7)	4·6 (3·7 to 5·3)	27·7 (18·2 to 37·4)	24·6 (16·4 to 32·9)	−7·5 (−9·0 to −5·8)
Americas	160 207 (86 968 to 235 078)	2·6 (1·4 to 3·9)	2·7 (1·5 to 4·0)	3·0 (1·6 to 4·6)	18·2 (9·8 to 26·7)	18·1 (9·9 to 26·4)	1·0 (−1·5 to 4·5)
Northern America	86 097 (50 232 to 122 855)	3·1 (1·8 to 4·4)	3·1 (1·8 to 4·5)	2·8 (1·3 to 4·4)	25·9 (15·2 to 36·9)	26·0 (15·1 to 37·1)	1·4 (−1·3 to 4·0)
Latin America and the Caribbean	74 110 (36 736 to 112 222)	2·3 (1·1 to 3·5)	2·3 (1·2 to 3·5)	3·3 (1·8 to 4·8)	13·6 (6·6 to 20·6)	13·4 (6·8 to 20·2)	0·7 (−2·1 to 4·2)
Europe	127 890 (64 611 to 191 908)	1·5 (0·8 to 2·2)	1·7 (0·9 to 2·4)	4·4 (2·2 to 5·6)	17·7 (8·9 to 26·6)	17·4 (9·1 to 25·8)	−3·8 (−8·2 to −1·2)
Northern Europe	13 236 (8036 to 18 475)	1·4 (0·8 to 1·9)	1·6 (1·0 to 2·2)	4·2 (0·6 to 6·5)	14·2 (8·6 to 19·7)	13·2 (8·1 to 18·3)	−7·5 (−13·1 to −2·8)
Eastern Europe	55 396 (18 825 to 92 378)	1·3 (0·4 to 2·1)	1·4 (0·5 to 2·3)	2·6 (0·7 to 4·0)	18·9 (6·5 to 31·4)	17·7 (6·4 to 29·2)	−8·3 (−11·4 to −5·5)
Western Europe	29 868 (17 884 to 42 046)	1·6 (0·9 to 2·2)	1·8 (1·1 to 2·5)	4·5 (−0·2 to 6·9)	15·0 (9·0 to 21·2)	17·3 (10·4 to 24·3)	5·3 (−0·1 to 9·5)
Southern Europe	29 390 (19 867 to 39 009)	2·1 (1·4 to 2·8)	2·2 (1·5 to 2·9)	2·9 (0·8 to 5·5)	20·7 (13·9 to 27·5)	19·7 (13·3 to 26·2)	−2·0 (−6·7 to 3·1)
Africa	170 094 (50 522 to 291 300)	1·7 (0·5 to 2·9)	1·8 (0·5 to 3·0)	3·3 (1·9 to 4·6)	22·2 (6·3 to 38·4)	12·9 (3·9 to 22·0)	−31·3 (−32·7 to −29·6)
Northern Africa	42 326 (28 057 to 56 784)	3·6 (2·4 to 4·8)	3·6 (2·4 to 4·8)	0·7 (−1·1 to 2·3)	23·6 (15·6 to 31·7)	19·0 (12·6 to 25·5)	−12·2 (−13·4 to −10·7)
Sub-Saharan Africa	127 768 (22 465 to 234 516)	1·4 (0·2 to 2·6)	1·4 (0·2 to 2·7)	0·2 (−1·1 to 1·6)	21·9 (3·8 to 40·1)	11·4 (1·8 to 21·2)	−373 (−39·0 to −35·6)
Asia	1 288 284 (955 630 to 1 627 654)	4·5 (3·4 to 5·7)	4·7 (3·5 to 6·0)	2·1 (1·0 to 3·0)	33·1 (24·6 to 41·7)	30·5 (22·5 to 38·6)	−5·3 (−6·9 to −3·6)
Southeast Asia	113 360 (74 167 to 153 030)	3·0 (1·9 to 4·0)	3·3 (2·2 to 4·5)	2·8 (0·6 to 4·8)	20·7 (13·7 to 27·8)	19·3 (12·6 to 26·1)	−9·8 (−13·2 to −4·8)
Western Asia	63 169 (51 417 to 75 071)	5·5 (4·5 to 6·5)	5·7 (4·7 to 6·8)	3·2 (1·7 to 5·4)	32·2 (26·3 to 38·2)	25·7 (20·9 to 30·6)	−10·9 (−13·9 to −7·8)
Central Asia	19 959 (14 820 to 25 190)	5·1 (3·8 to 6·4)	5·1 (3·8 to 6·4)	0·7 (−0·5 to 2·5)	37·6 (28·0 to 47·3)	28·2 (20·9 to 35·6)	−14·9 (−17·1 to −13·4)
Southern Asia	646 213 (483 012 to 812 162)	5·2 (3·9 to 6·6)	5·2 (3·9 to 6·6)	1·1 (−0·4 to 2·4)	44·0 (32·9 to 55·4)	34·8 (26·0 to 43·8)	−12·5 (−15·2 to −10·2)
Eastern Asia	445 583 (332 214 to 562 201)	4·1 (3·1 to 5·1)	4·5 (3·3 to 5·7)	3·1 (1·5 to 4·4)	26·9 (20·3 to 33·6)	30·7 (22·8 to 38·9)	6·2 (4·7 to 7·2)
Oceania	6917 (2171 to 11 778)	2·9 (0·7 to 5·2)	3·6 (1·2 to 6·1)	8·7 (6·1 to 11·6)	21·1 (5·4 to 37·3)	21·5 (7·3 to 36·2)	−4·4 (−9·5 to 1·9)
Australia and New Zealand	6184 (2478 to 9980)	3·4 (1·3 to 5·4)	4·3 (1·7 to 6·9)	7·3 (4·3 to 10·4)	22·7 (9·1 to 36·6)	26·3 (10·6 to 42·4)	0·7 (−3·7 to 6·7)
Other regions in Oceania	733 (−308 to 1798)	1·8 (−0·8 to 4·4)	1·3 (−0·6 to 3·3)	−5·8 (−18·4 to 5·6)	15·9 (−6·9 to 39·6)	6·9 (−2·9 to 17·0)	−37·4 (−52·5 to −23·9)

All regions in the table are defined according to UN Statistics Division (M49) regional groupings. Other regions in Oceania are defined as all areas outside Australia and New Zealand in Oceania. Corresponding scatter plots from 2000 and 2019 are shown in the appendix (pp 17–20).

* Percentage change per decade was estimated based on a linear regression model considering a Gaussian distribution of percentage excess and excess death rate on the log scale. The 95% CI of percentage change per decade was obtained based on 1000 bootstrap replicates.

## Data Availability

Data were collected within the Multi-Country Multi-City (MCC) Collaborative Research Network under a data sharing agreement and cannot be made publicly available. Researchers can refer to MCC participants, who are listed as coauthors of this Article, for information on accessing the data for each country.

## Research in context

### Evidence before this study

In the past few years, attention has been increasingly paid to the negative health effects of short-term temperature variability, an indicator of unstable weather that causes challenges to human adaption. We searched MEDLINE (via PubMed), Web of Science, and Google Scholar from database inception until Nov 6, 2021, for articles in English. We used a combination of search terms, including exposure terms (“temperature variability”, “temperature change”, “temperature fluctuation”, “temperature range”) and health outcome terms (“mortality”, “mortality burden”, “death”, “excess death”). Although many studies have reported the association between temperature variability and premature deaths, most evidence has been obtained from studies in single cities or countries; the systematic evaluation of results is challenged by differences in modelling, parameterisation, and publication bias. One study evaluated the mortality risk associated with temperature variability based on data from 12 countries but did not provide the attributable burden of temperature variability-related mortality. The global burden of mortality associated with temperature variability remains unknown.

### Added value of this study

To our knowledge, this is the largest and first study, using global gridded observation data at a spatial resolution of 0·5° × 0·5°, to systematically estimate the global burden of mortality associated with temperature variability and explore its temporal trend over 20 years. This study identified an increasing trend of temperature variability at the global level from 2000–19. Globally, 1 753 392 deaths were associated with temperature variability per year, accounting for 3·4% of all deaths. The percentage excess in mortality increased about 4·6% per decade from 2000–19. Disparate geographical variations were also found.

### Implications of all the available evidence

This study provides robust epidemiological evidence of the impact of temperature variability on mortality globally, and across and within countries or regions. Our findings suggest that regions with a higher percentage excess in mortality due to temperature variability (eg, the whole of Asia, Australia and New Zealand, and northern Africa) are of great importance to contribute to coordinated actions for health, and more targeted policies should be implemented in regions with a higher increasing rate of temperature variability-related mortality burden (eg, Australia and New Zealand, Europe, and the Americas).
