# Supplementary material for: Global, regional, and national burden of mortality associated with short-term temperature variability from 2000–19: a three-stage modelling study
Source: Lancet Planet Health. Author manuscript; Available in PMC 2022 Jun 8. (PMC9177161; doi:10.1016/S2542-5196(22)00073-0)
Supplement: 1 [file NIHMS1805417-supplement-1.pdf]

THE LANCET
Planetary Health

Supplementary appendix

This appendix formed part of the original submission and has been peer reviewed. We post it as supplied by the authors.

Supplement to: Wu Y, Li S, Zhao Q, et al. Global, regional, and national burden of mortality associated with short-term temperature variability from 2000–19: a three-stage modelling study. *Lancet Planet Health* 2022; **6:** e409–20.

Global, regional, and national burden of mortality associated with short-term temperature variability from 2000 to 2019: a three-stage modelling study

Supplementary Appendix

Table of contents

Text S1. e Methods.
Table S1. Descriptive statistics by country/region included in MCC study.
Table S2. Missing rates by country/region included in MCC study.
Table S3. Meta-regression models for explaining variation in overall TV effects: Cochran Q test for heterogeneity, I^2 statistics for residual heterogeneity.
Table S4. The average annual daily temperature variability in 2000 and 2019 by continent and region.
Figure S1. The long-term trend of temperature variability after seasonal-trend decomposition by region from 2000 to 2019.
Table S5. Percentage change in mortality associated with an interquartile increase in temperature variability by country.
Table S6. Average annual percentage excess in mortality and excess deaths per 100,000 residents due to temperature variability in 2000–19 by continent and region.
Table S7. Average annual percentage excess in mortality and excess deaths per 100,000 residents due to temperature variability between 2000–19 by the indicators of Köppen-Geiger climate classification.
Figure S2. Scatter plots of percentage excess in mortality associated with temperature variability from 2000 to 2019.
Figure S3. Scatter plots of excess death rate associated with temperature variability from 2000 to 2019.
Figure S4. Leading 20 countries of excess deaths in 2000 and 2019.
Table S8. Average annual global percentage excess in mortality and global excess deaths per 100,000 residents due to temperature variability in 2000–19 on different exposure days.
Table S9. Results of sensitivity analyses on global percentage excess in mortality and global excess deaths per 100,000 residents.
Table S10. Results of sensitivity analyses on overall TV-mortality association based on 500 locations with relative humidity data.
Table S11. Average annual percentage excess in mortality under the counterfactual scenario of grid-specific mean temperature variability by continent and region.

Text S1. eMethods

Sensitivity analyses

The range of each parameter was decided based on prior knowledge and research.
(1) A maximum exposure period of seven days for short-term TV exposure is commonly used in prior research [1-5]**.** However, the use of a single length of exposure is insufficient to provide evidence on the short-term impact of TV, thus we use alternative lag from 1 to 6 days and from 8 to 10 days to check if the association still exists.
(2) A lag period of 21 days for mean temperature was commonly used to include the long delay of the effects of cold temperatures [6]. Here, we used longer lag days (24 or 28 lag days) in the sensitivity analyses in case a lag of 21 days was not enough to capture the temperature effects on mortality.
(3) We chose the most commonly used value of the degree of freedom in the main analysis and applied neighboring values on each side to make sure that our results can exist independently of any particular. Small degrees of freedom will fail to capture the main long-term patterns closely, whereas too many will result in overfitting—that a very 'wobbly' function which may compete with the variable of interest to explain the short-term variation of interest, widening confidence intervals of relative risk estimates [7].
(4) We adjusted relative humidity in the first stage using data from 500 locations with relative humidity data. As 500 locations failed to cover Africa and areas with polar and alpine climates, we were unable to estimate the global mortality burden associated with TV using data from these locations. Thus, we compared the TV-mortality associations with and without adjustment of relative humidity in the sensitivity analyses.

Table S1. Descriptive statistics by country/region included in MCC study.

Country/Region	Study period	No. of locations	Total deaths
Americas			
Argentina	2005-2015	3	686,333
Brazil	1997-2011	18	3,258,661
Canada	1986-2015	26	3,733,749
Chile	2004-2014	4	285,776
Colombia	1998-2013	5	956,539
Costa Rica	2000-2017	1	31,117
Ecuador	2014-2018	2	112,264
Guatemala	2009-2016	1	62,715
Mexico	1998-2014	10	2,980,086
Panama	2013-2016	1	11,457
Paraguay	2004-2016	1	39,713
Peru	2008-2014	18	628,420
Puerto Rico	2009-2016	1	26,564
United States	1979-2006	211	32,089,970
Uruguay	2012-2016	1	153,554
Europe			
Czech Republic	1994-2015	4	711,910
Estonia	1997-2015	5	146,347
Finland	1994-2014	1	153,308
France	2000-2014	18	1,639,262
Germany	1993-2015	12	3,105,865
Greece	2001-2010	1	287,969
Ireland	1984-2007	6	1,058,215
Italy	2006-2015	18	801,302
Moldova	2001-2010	4	59,906
Netherlands	1995-2016	5	453,395
Norway	1979-2016	1	204,188
Portugal	1980-2018	5	1,750,670
Romania	1994-2016	8	951,146
Spain	1990-2014	52	2,809,154
Sweden	1990-2016	3	717,294
Switzerland	1995-2013	8	243,638
United Kingdom	1990-2016	70	6,064,610
Africa			
South Africa	1997-2013	52	8,509,130
Asia			
China	1996-2015	15	896,223
Iran, Islamic Rep.	2004-2013	1	121,585
Japan	1972-2015	47	35,214,892
Korea, Rep.	1997-2018	36	3,070,357

Kuwait	2000-2016	1	73,748
Philippines	2006-2010	4	274,516
Taiwan	1994-2014	3	1,209,573
Thailand	1999-2008	62	1,827,853
Vietnam	2009-2013	2	108,173
Oceania			
Australia	1988-2009	3	1,177,950

Table S2. Missing rates by country/region included in MCC study.

Country/Region	Missing rate (%)			
	Total	Death counts	Minimum temperature	Maximum temperature
Argentina	0.30	0.30	0.00	0.00
Australia	0.17	0.16	0.01	0.01
Brazil	0.92	0.00	0.90	0.71
Canada	1.36	0.88	0.47	0.47
Chile	0.05	0.05	0.00	0.00
China	0.43	0.43	0.00	0.00
Colombia	0.51	0.01	0.47	0.04
Costa Rica	0.00	0.00	0.00	0.00
Czech Republic	0.00	0.00	0.00	0.00
Ecuador	0.00	0.00	0.00	0.00
Estonia	0.00	0.00	0.00	0.00
Finland	0.01	0.01	0.00	0.00
France	0.18	0.18	0.00	0.00
Germany	0.01	0.01	0.00	0.00
Greece	5.97	0.00	5.97	5.91
Guatemala	0.00	0.00	0.00	0.00
Iran	2.11	2.11	0.00	0.00
Ireland	0.20	0.01	0.19	0.19
Italy	0.68	0.00	0.58	0.34
Japan	0.17	0.00	0.16	0.16
Kuwait	0.00	0.00	0.00	0.00
Mexico	0.11	0.11	0.00	0.00
Moldova	0.00	0.00	0.00	0.00
Netherland	0.00	0.00	0.00	0.00
Norway	1.28	1.11	0.17	0.17
Panama	0.00	0.00	0.00	0.00
Paraguay	0.00	0.00	0.00	0.00
Peru	2.32	1.49	0.73	0.83
Philippines	0.14	0.14	0.00	0.00
Portugal	0.24	0.08	0.17	0.17
Puerto Rico	0.00	0.00	0.00	0.00
Romania	0.00	0.00	0.00	0.00
South Africa	0.09	0.00	0.09	0.02
South Korea	0.00	0.00	0.00	0.00
Spain	0.01	0.00	0.01	0.01
Sweden	0.01	0.00	0.01	0.01
Switzerland	0.00	0.00	0.00	0.00
Taiwan	0.03	0.02	0.01	0.01
Thailand	0.41	0.00	0.41	0.19
UK	0.01	0.00	0.01	0.01

Uruguay	0.00	0.00	0.00	0.00
USA	0.29	0.00	0.29	0.29
Vietnam	0.00	0.00	0.00	0.00
**Global**	**0.31**	**0.09**	**0.22**	**0.18**

Table S3. Meta-regression models for explaining variation in overall TV effects: Cochran Q test for heterogeneity, I^2 statistics for residual heterogeneity.

Predictor	I-square	P-value for Q test
Köppen climate classification	36.71	< 0.001
Region	29.77	< 0.001
Yearly average of daily mean temperature	34.63	< 0.001
Range of daily mean temperature	26.53	< 0.001
GDP per capita	29.68	< 0.001
All predictors (final model)	22.67	< 0.001

Table S4. The average annual daily temperature variability in 2000 and 2019 by continent and region.

Region	Mean ± SD (°C)	
	2000	2019
Global	6.0 ± 1.3	6.2 ± 1.3
Americas	6.4 ± 1.4	6.4 ± 1.4
Northern America	7.0 ± 1.3	7.0 ± 1.3
Latin America and the Caribbean	5.8 ± 1.3	5.9 ± 1.4
Europe	5.5 ± 1.1	5.8 ± 1.1
Northern Europe	4.3 ± 0.7	4.7 ± 0.8
Eastern Europe	5.8 ± 0.9	6.1 ± 1.0
Western Europe	4.7 ± 0.6	5.2 ± 0.7
Southern Europe	5.6 ± 0.9	5.8 ± 1.0
Africa	6.1 ± 1.3	6.2 ± 1.5
Northern Africa	6.9 ± 0.9	6.9 ± 1.1
Sub-Saharan Africa	5.9 ± 1.2	6.0 ± 1.5
Asia	6.1 ± 1.3	6.3 ± 1.2
South-eastern Asia	4.5 ± 0.9	5.0 ± 1.0
Western Asia	6.9 ± 1.2	6.7 ± 0.9
Central Asia	6.7 ± 0.7	6.9 ± 0.6
Southern Asia	6.2 ± 1.0	6.4 ± 1.1
Eastern Asia	6.2 ± 1.3	6.4 ± 1.3
Oceania	5.2 ± 1.2	5.5 ± 1.6
Australia and New Zealand	5.1 ± 1.0	6.1 ± 1.3
Other regions in Oceania	5.5 ± 1.7	4.1 ± 1.4

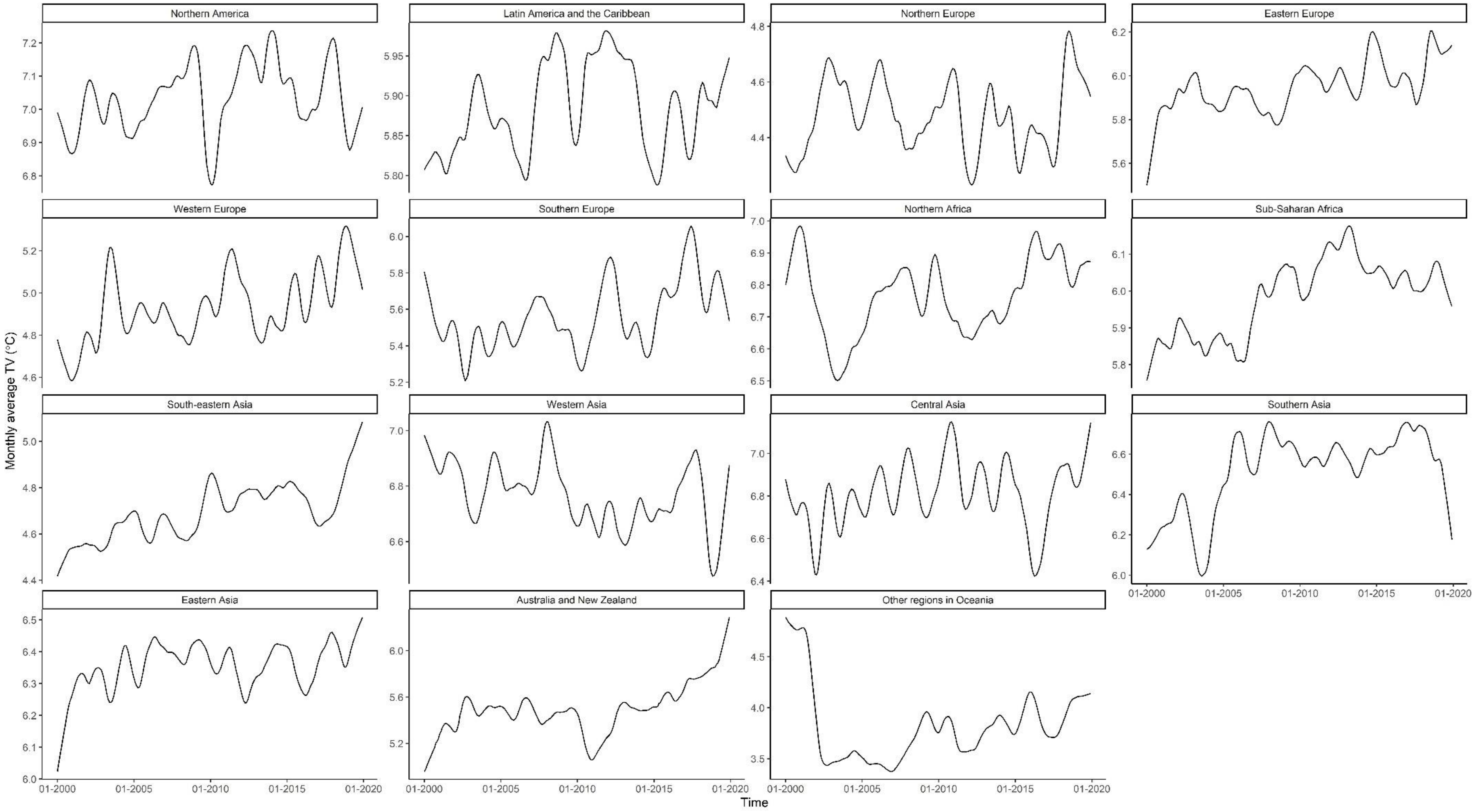

Figure S1. The long-term trend of temperature variability after seasonal-trend decomposition by region from 2000 to 2019.

Table S5. Percentage change in mortality associated with an interquartile increase in temperature variability.

Country/Region	Percentage change in mortality (%)
Afghanistan	1.70 (1.66 to 1.74)
Albania	0.65 (0.61 to 0.70)
Algeria	0.69 (0.68 to 0.71)
Angola	0.49 (0.44 to 0.54)
Antigua and Barbuda	0.16 (0.09 to 0.22)
Argentina	0.85 (0.84 to 0.86)
Armenia	1.39 (1.31 to 1.48)
Australia	1.16 (1.12 to 1.20)
Austria	0.60 (0.57 to 0.64)
Azerbaijan	1.46 (1.39 to 1.53)
The Bahamas	0.23 (0.20 to 0.26)
Bahrain	1.62 (1.40 to 1.83)
Bangladesh	1.57 (1.48 to 1.66)
Barbados	0.19 (0.09 to 0.30)
Belarus	0.63 (0.60 to 0.66)
Belgium	0.70 (0.65 to 0.74)
Belize	0.25 (0.20 to 0.29)
Benin	0.45 (0.34 to 0.56)
Bhutan	1.85 (1.75 to 1.95)
Bolivia	0.77 (0.72 to 0.82)
Bosnia and Herzegovina	0.59 (0.55 to 0.63)
Botswana	1.41 (1.38 to 1.44)
Brazil	0.45 (0.44 to 0.46)
Brunei Darussalam	0.38 (0.33 to 0.43)
Bulgaria	0.57 (0.53 to 0.61)
Burkina Faso	1.25 (1.13 to 1.38)
Burundi	0.15 (0.02 to 0.27)
Cabo Verde	0.08 (0.02 to 0.13)
Cambodia	1.01 (0.96 to 1.06)
Cameroon	0.23 (0.19 to 0.27)
Canada	0.34 (0.33 to 0.34)
Central African Republic	0.39 (0.32 to 0.46)
Chad	1.26 (1.22 to 1.31)
Chile	0.59 (0.57 to 0.61)
China	1.14 (1.13 to 1.15)
Colombia	0.25 (0.24 to 0.27)
Comoros	0.17 (0.06 to 0.27)
Democratic Republic of the Congo	0.14 (0.12 to 0.15)
Republic of the Congo	0.11 (0.09 to 0.13)
Costa Rica	0.35 (0.30 to 0.40)
Côte d'Ivoire	0.19 (0.16 to 0.23)

Croatia	0.57 (0.53 to 0.61)
Cuba	0.22 (0.20 to 0.25)
Cyprus	0.87 (0.78 to 0.96)
Czech Republic	0.65 (0.62 to 0.69)
Denmark	-0.22 (-0.34 to -0.10)
Djibouti	0.72 (0.63 to 0.80)
Dominican Republic	0.25 (0.21 to 0.30)
Ecuador	0.38 (0.32 to 0.44)
The Arab Republic of Egypt	0.64 (0.63 to 0.65)
El Salvador	0.42 (0.33 to 0.52)
Equatorial Guinea	0.14 (0.08 to 0.20)
Eritrea	0.63 (0.59 to 0.67)
Estonia	0.44 (0.40 to 0.48)
Eswatini	0.89 (0.77 to 1.00)
Ethiopia	0.63 (0.60 to 0.66)
Fiji	0.37 (0.31 to 0.44)
Finland	0.35 (0.33 to 0.37)
France	0.54 (0.53 to 0.56)
Gabon	0.10 (0.08 to 0.12)
The Gambia	1.93 (1.58 to 2.28)
Georgia	1.15 (1.11 to 1.20)
Germany	0.66 (0.64 to 0.68)
Ghana	0.17 (0.13 to 0.21)
Greece	0.54 (0.50 to 0.57)
Grenada	0.20 (0.12 to 0.27)
Guatemala	0.45 (0.39 to 0.51)
Guinea	0.23 (0.18 to 0.28)
Guinea-Bissau	0.34 (0.22 to 0.46)
Guyana	0.21 (0.19 to 0.23)
Haiti	0.19 (0.15 to 0.23)
Honduras	0.24 (0.21 to 0.27)
Hungary	0.69 (0.65 to 0.72)
Iceland	0.22 (0.18 to 0.26)
India	2.19 (2.14 to 2.24)
Indonesia	0.49 (0.48 to 0.49)
The Islamic Republic of Iran	1.61 (1.57 to 1.64)
Iraq	1.92 (1.87 to 1.97)
Ireland	0.34 (0.32 to 0.35)
Israel	1.36 (1.26 to 1.47)
Italy	0.50 (0.48 to 0.52)
Jamaica	0.15 (0.11 to 0.19)
Japan	0.86 (0.84 to 0.88)
Jordan	1.55 (1.48 to 1.61)
Kazakhstan	1.38 (1.36 to 1.40)

Kenya	0.60 (0.56 to 0.65)
Kiribati	0.55 (0.34 to 0.76)
North Korea	1.01 (0.98 to 1.03)
The Republic of Korea	1.07 (1.03 to 1.11)
Kuwait	1.81 (1.72 to 1.91)
Kyrgyz Republic	1.28 (1.19 to 1.38)
Lao People's Democratic Republic	1.43 (1.34 to 1.53)
Latvia	0.50 (0.46 to 0.55)
Lebanon	1.29 (1.13 to 1.45)
Lesotho	0.50 (0.43 to 0.56)
Liberia	0.21 (0.15 to 0.27)
Libya	0.73 (0.71 to 0.75)
Liechtenstein	0.46 (0.15 to 0.77)
Lithuania	0.62 (0.57 to 0.68)
Luxembourg	0.73 (0.62 to 0.83)
Madagascar	0.32 (0.29 to 0.36)
Malawi	0.44 (0.37 to 0.51)
Malaysia	0.38 (0.37 to 0.40)
Maldives	0.71 (0.65 to 0.76)
Mali	1.28 (1.22 to 1.33)
Malta	0.14 (-0.03 to 0.32)
Mauritania	1.08 (1.04 to 1.13)
Mauritius	0.09 (-0.05 to 0.23)
Mexico	0.99 (0.96 to 1.02)
Federated States of Micronesia	0.64 (0.49 to 0.79)
Moldova	0.58 (0.52 to 0.64)
Mongolia	0.93 (0.91 to 0.95)
Montenegro	0.61 (0.54 to 0.68)
Morocco	0.63 (0.61 to 0.66)
Mozambique	0.41 (0.36 to 0.46)
Myanmar	1.89 (1.81 to 1.97)
Namibia	1.11 (1.07 to 1.15)
Nepal	1.83 (1.71 to 1.96)
Netherlands	0.55 (0.49 to 0.62)
New Zealand	0.62 (0.59 to 0.66)
Nicaragua	0.25 (0.22 to 0.28)
Niger	1.23 (1.19 to 1.28)
Nigeria	0.72 (0.64 to 0.81)
North Macedonia	0.71 (0.64 to 0.77)
Norway	0.31 (0.30 to 0.33)
Oman	1.47 (1.38 to 1.57)
Pakistan	1.90 (1.84 to 1.97)
Panama	0.29 (0.26 to 0.32)
Papua New Guinea	0.34 (0.31 to 0.37)

Paraguay	0.82 (0.76 to 0.88)
Peru	0.48 (0.46 to 0.51)
Philippines	0.48 (0.47 to 0.49)
Poland	0.60 (0.57 to 0.62)
Portugal	0.81 (0.71 to 0.90)
Qatar	1.60 (1.42 to 1.78)
Romania	0.58 (0.55 to 0.60)
Russian Federation	0.24 (0.24 to 0.25)
Rwanda	0.27 (0.16 to 0.39)
Sao Tome and Principe	0.08 (0.00 to 0.17)
Saudi Arabia	1.21 (1.20 to 1.23)
Senegal	1.41 (1.28 to 1.54)
Serbia	0.67 (0.63 to 0.71)
Seychelles	0.16 (0.08 to 0.23)
Sierra Leone	0.18 (0.12 to 0.24)
Singapore	0.49 (0.28 to 0.70)
Slovak Republic	0.70 (0.65 to 0.75)
Slovenia	0.64 (0.59 to 0.69)
Solomon Islands	0.26 (0.22 to 0.30)
Somalia	0.76 (0.74 to 0.78)
South Africa	0.79 (0.77 to 0.82)
Spain	0.75 (0.71 to 0.78)
Sri Lanka	0.66 (0.61 to 0.70)
St. Lucia	0.20 (0.12 to 0.28)
St. Vincent and the Grenadines	0.17 (0.07 to 0.26)
Sudan	1.00 (0.97 to 1.03)
Suriname	0.27 (0.23 to 0.30)
Sweden	0.38 (0.37 to 0.40)
Switzerland	0.54 (0.49 to 0.60)
Syrian Arab Republic	1.78 (1.70 to 1.85)
Tajikistan	1.72 (1.61 to 1.82)
Tanzania	0.25 (0.22 to 0.28)
Thailand	1.13 (1.07 to 1.20)
Timor-Leste	0.97 (0.88 to 1.06)
Togo	0.24 (0.17 to 0.31)
Trinidad and Tobago	0.21 (0.14 to 0.28)
Tunisia	0.74 (0.70 to 0.78)
Turkey	1.40 (1.36 to 1.43)
Turkmenistan	1.80 (1.76 to 1.83)
Uganda	0.18 (0.13 to 0.22)
Ukraine	0.56 (0.54 to 0.57)
United Arab Emirates	1.67 (1.63 to 1.72)
United Kingdom	0.38 (0.37 to 0.40)
United States	0.65 (0.64 to 0.65)

Uruguay	0.77 (0.74 to 0.79)
Uzbekistan	1.81 (1.77 to 1.85)
Vanuatu	0.49 (0.40 to 0.57)
The Bolivarian Republic of Venezuela	0.25 (0.24 to 0.27)
Vietnam	0.79 (0.74 to 0.85)
West Bank and Gaza	1.32 (1.15 to 1.50)
The Republic of Yemen	1.34 (1.30 to 1.37)
Zambia	0.80 (0.74 to 0.86)
Zimbabwe	1.10 (1.05 to 1.15)

Table S6. Average annual percentage excess in mortality and excess deaths per 100,000 residents due to temperature variability in 2000–19 by continent and region.

Country/Region	Percentage excess in mortality (%)	Excess deaths per 100,000 residents
Global	3.4 (2.2 to 4.6)	26 (17 to 35)
Americas	2.7 (1.5 to 4.0)	17 (9 to 25)
Northern America	3.2 (1.8 to 4.5)	25 (15 to 36)
Latin America and the Caribbean	2.3 (1.1 to 3.5)	13 (6 to 19)
Europe	1.6 (0.8 to 2.3)	17 (9 to 26)
Northern Europe	1.5 (0.9 to 2.1)	13 (8 to 19)
Eastern Europe	1.3 (0.5 to 2.2)	19 (6 to 31)
Western Europe	1.7 (1.0 to 2.4)	16 (9 to 22)
Southern Europe	2.1 (1.4 to 2.8)	19 (13 to 25)
Africa	1.7 (0.5 to 3.0)	17 (5 to 30)
Northern Africa	3.6 (2.4 to 4.9)	21 (14 to 28)
Sub-Saharan Africa	1.5 (0.3 to 2.7)	16 (3 to 30)
Asia	4.7 (3.5 to 5.9)	31 (23 to 40)
South-eastern Asia	3.2 (2.1 to 4.3)	19 (12 to 26)
Western Asia	5.7 (4.6 to 6.7)	28 (23 to 33)
Central Asia	5.1 (3.8 to 6.5)	33 (25 to 42)
Southern Asia	5.3 (4.0 to 6.7)	39 (29 to 48)
Eastern Asia	4.3 (3.2 to 5.5)	29 (22 to 37)
Oceania	3.2 (1.0 to 5.4)	20 (6 to 34)
Australia and New Zealand	3.8 (1.5 to 6.1)	23 (9 to 38)
Other regions in Oceania	1.3 (-0.6 to 3.3)	9 (-4 to 22)

Table S7. Average annual percentage excess in mortality and excess deaths per 100,000 residents due to temperature variability between 2000–19 by the indicators of Köppen-Geiger climate classification.

Climate classification	Percentage excess in mortality (%)	Excess deaths per 100,000 residents
Group A: Tropical climates	2.1 (0.9 to 3.4)	16 (7 to 25)
Group B: Dry climates	6.0 (4.6 to 7.5)	43 (33 to 54)
Group C: Temperature climates	3.4 (2.5 to 4.3)	26 (19 to 33)
Group D: Continental climates	2.3 (1.4 to 3.3)	22 (13 to 32)
Group E: Polar and alpine climates	-2.6 (-9.3 to 4.6)	-18 (-65 to 33)

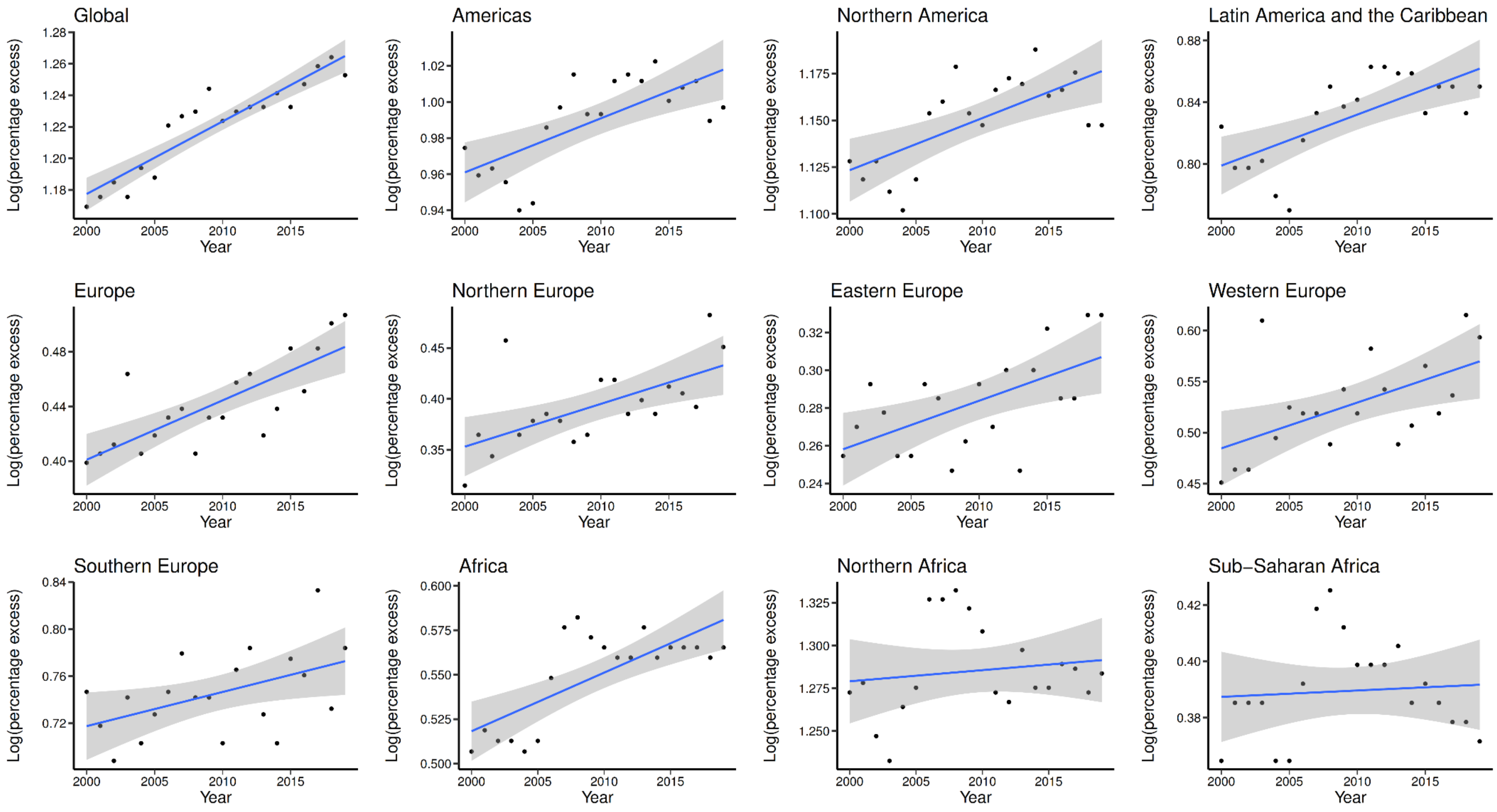
Global
Americas
Northern America
Latin America and the Caribbean
Europe
Northern Europe
Eastern Europe
Western Europe
Southern Europe
Africa
Northern Africa
Sub−Saharan Africa
Log(percentage excess)
Year

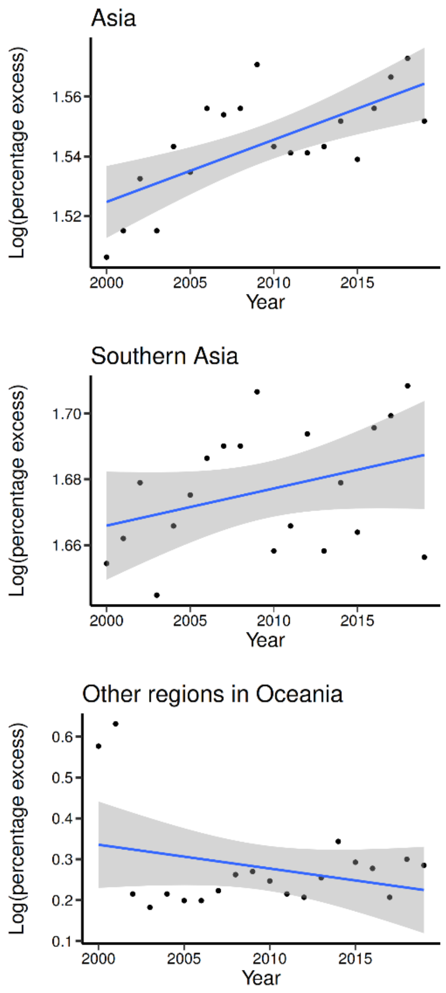

Figure S2. Scatter plots of percentage excess in mortality associated with temperature variability from 2000 to 2019.

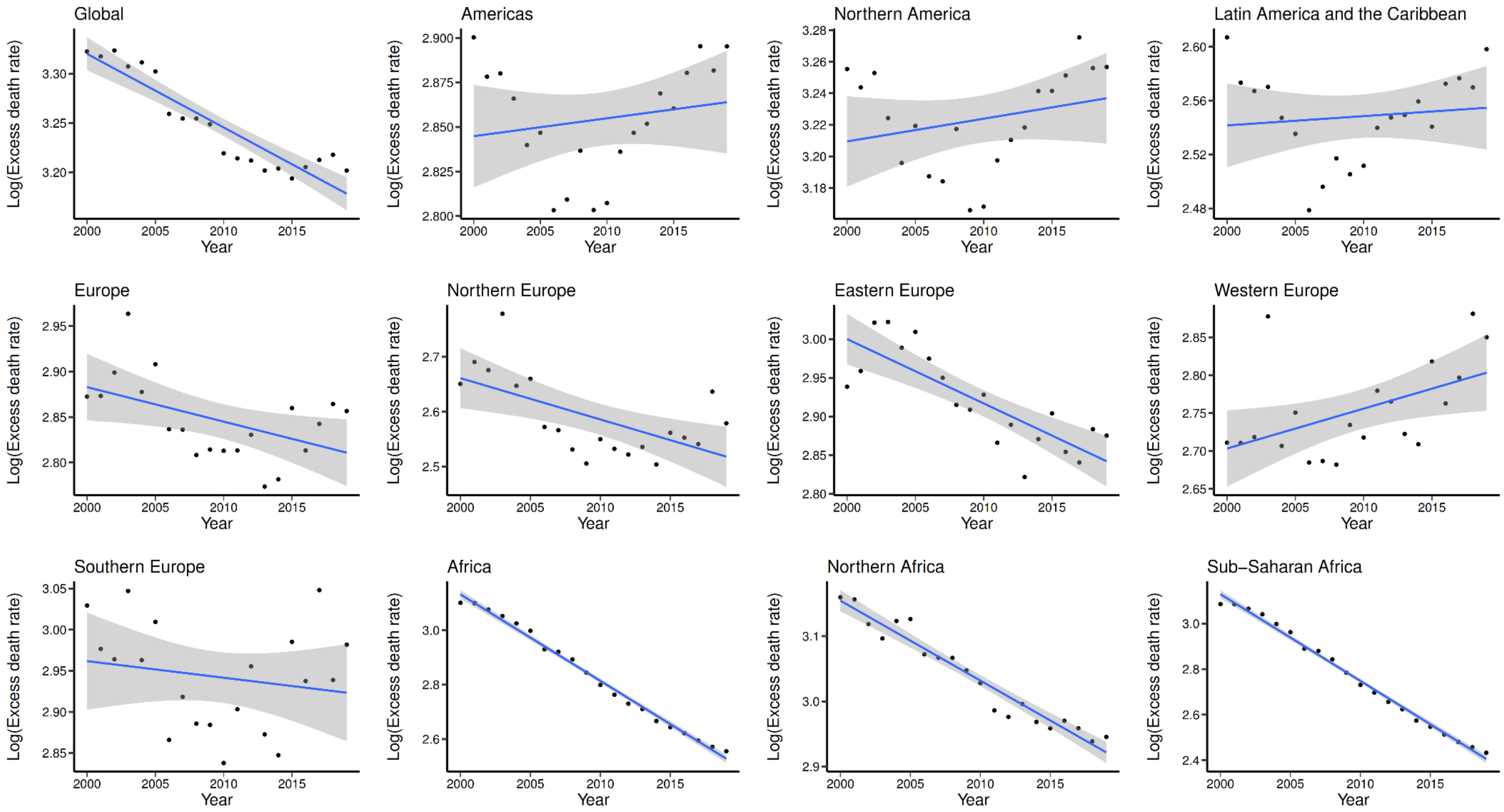

Global
Americas
Northern America
Latin America and the Caribbean
Europe
Northern Europe
Eastern Europe
Western Europe
Southern Europe
Africa
Northern Africa
Sub−Saharan Africa
Log(Excess death rate)
Year

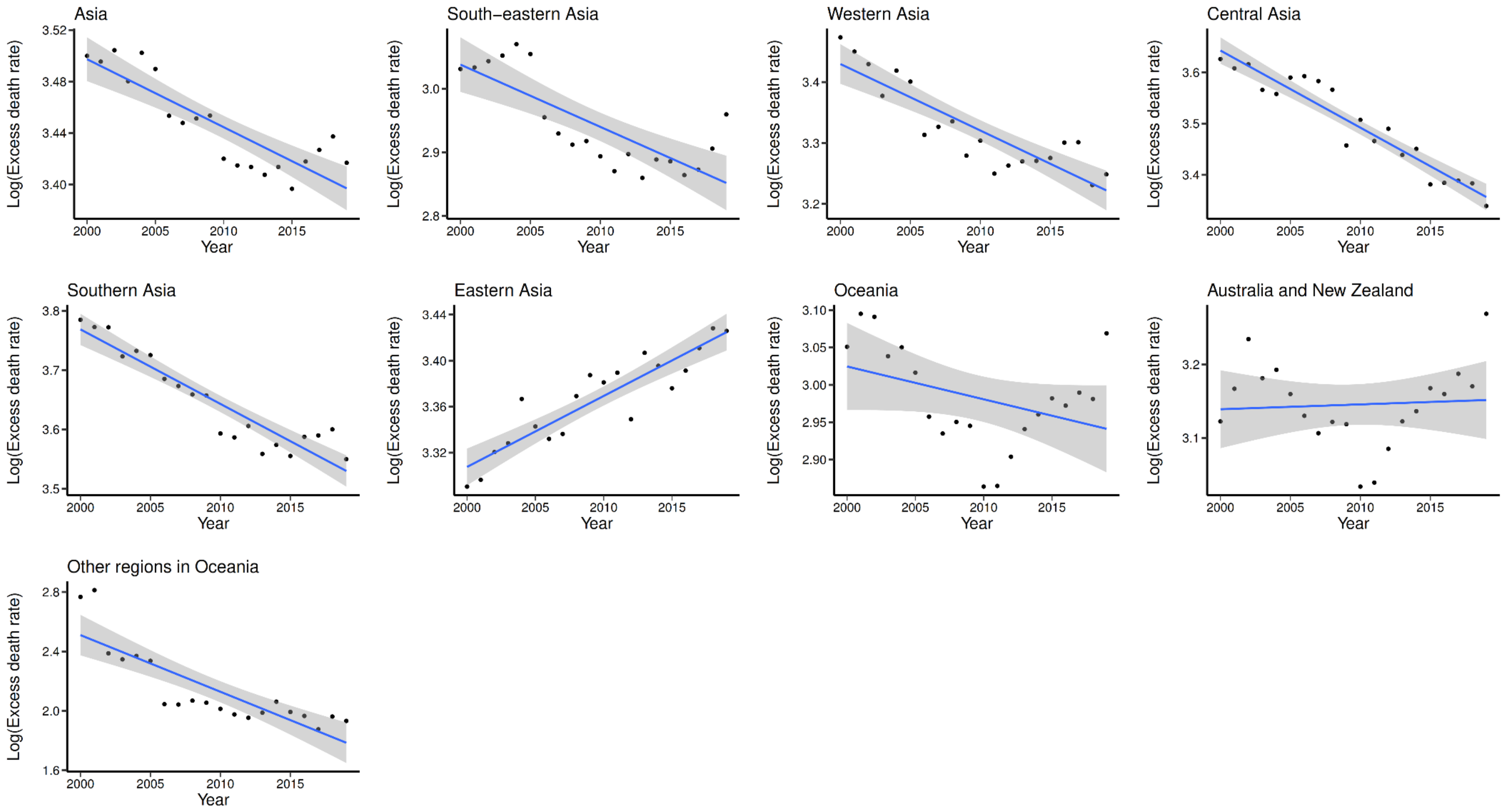

Figure S3. Scatter plots of excess death rate associated with temperature variability from 2000 to 2019.

Country	Excess deaths in 2000
1 India	498,171 (369,887 to 628,652)
2 China	333,967 (252,328 to 417,660)
3 Pakistan	78,735 (62,748 to 94,947)
4 United States	77,861 (46,087 to 110,417)
5 Indonesia	43,903 (28,156 to 59,806)
6 Japan	41,437 (29,993 to 53,158)
7 Russian Federation	29,652 (5,542 to 54,066)
8 Bangladesh	27,872 (18,008 to 37,846)
9 Brazil	22,650 (11,484 to 33,950)
10 Myanmar	22,119 (15,579 to 28,746)
11 Ethiopia	21,518 (11,030 to 32,131)
12 Turkey	19,647 (16,509 to 22,813)
13 Nigeria	19,476 (-8,904 to 48,276)
14 Mexico	19,410 (13,389 to 25,507)
15 Iran, Islamic Rep.	18,742 (14,892 to 22,643)
16 Egypt, Arab Rep.	16,882 (11,524 to 22,313)
17 Thailand	15,805 (9,583 to 22,129)
18 South Africa	15,608 (9,078 to 22,225)
19 Vietnam	14,175 (10,245 to 18,139)
20 Germany	13,022 (8,169 to 17,919)
22 Sudan	11,335 (7,561 to 15,169)
25 Philippines	10,612 (6,918 to 14,337)

Country	Excess deaths in 2019
1 India	49,0478 (363,353 to 619,705)
2 China	407,791 (303,605 to 515,242)
3 United States	91,922 (54,090 to 130,722)
4 Pakistan	88,935 (70,816 to 107,330)
5 Japan	56,782 (40,479 to 73,516)
6 Indonesia	46,284 (29,482 to 63,294)
7 Bangladesh	28,699 (18,519 to 39,005)
8 Mexico	27,282 (18,849 to 35,820)
9 Brazil	25,369 (12,956 to 37,925)
10 Iran, Islamic Rep.	24,237 (19,260 to 29,285)
11 Russian Federation	23,653 (4,506 to 43,065)
12 Thailand	19,617 (11,874 to 27,489)
13 Egypt, Arab Rep.	18,520 (12,638 to 24,479)
14 Turkey	18,325 (15,345 to 21,329)
15 Nigeria	17,422 (-7,684 to 42,881)
16 Myanmar	17,205 (11,906 to 22,583)
17 Sudan	16,282 (10,809 to 21,840)
18 Vietnam	16,153 (11,556 to 20,791)
19 Germany	15,963 (10,034 to 21,952)
20 Philippines	15,084 (9,840 to 20,380)
21 South Africa	14,902 (8,600 to 21,303)
22 Ethiopia	14,017 (7,054 to 21,064)

Figure S4. Leading 20 countries of excess deaths in 2000 and 2019.

Table S8. Average annual global percentage excess in mortality and global excess deaths per 100,000 residents due to temperature variability in 2000–19 on different exposure days.

Exposure days	Percentage excess in mortality (%)	P for difference	Excess death per 100,000 residents	P for difference
TV 0–1	2.5 (1.9 to 3.2)	0.21	19 (14 to 24)	0.21
TV 0–2	2.3 (1.6 to 3.1)	0.13	18 (12 to 24)	0.13
TV 0–3	2.4 (1.6 to 3.2)	0.17	18 (12 to 25)	0.17
TV 0–4	2.6 (1.7 to 3.5)	0.30	20 (13 to 27)	0.30
TV 0–5	2.9 (1.9 to 3.9)	0.55	22 (15 to 30)	0.55
TV 0–6	3.2 (2.1 to 4.3)	0.78	24 (16 to 32)	0.78
TV 0–7	3.4 (2.2 to 4.6)	Reference	26 (17 to 35)	Reference
TV 0–8	3.6 (2.4 to 4.8)	0.83	27 (18 to 37)	0.83
TV 0–9	3.6 (2.4 to 5.0)	0.77	28 (18 to 38)	0.77
TV 0–10	3.8 (2.4 to 5.2)	0.66	29 (19 to 39)	0.66

Table S9. Results of sensitivity analyses on global percentage excess in mortality and global excess deaths per 100,000 residents.

Parameters	Excess death ratio (%)	P for difference	Excess deaths per 100,000 residents	P for difference
Main model	3.4 (2.2 to 4.6)	Reference	26 (17 to 35)	Reference
Df/per year for seasonality and long-term trend				
6	3.5 (2.3 to 4.7)	0.91	27 (18 to 35)	0.91
8	3.5 (2.4 to 4.7)	0.86	27 (18 to 36)	0.86
Df for lag-response curve of temperature				
5	3.3 (2.2 to 4.5)	0.91	25 (17 to 34)	0.92
6	3.2 (2.1 to 4.4)	0.86	25 (16 to 33)	0.86
Lag period of temperature				
24	3.3 (2.1 to 4.4)	0.87	25 (16 to 34)	0.87
28	3.1 (2.0 to 4.3)	0.77	24 (15 to 33)	0.77
Different knots for temperature	3.5 (2.3 to 4.6)	0.91	27 (18 to 35)	0.91

Table S10. Results of sensitivity analyses on overall TV-mortality association based on 500 locations with relative humidity data.

Models	Coefficient	Standard error	P for difference
With relative humidity	0.0049	0.0002	Reference
Without relative humidity	0.0052	0.0002	0.29

Table S11. Average annual percentage excess in mortality under the counterfactual scenario of grid-specific minimum temperature variability by continent and region.

Region	Percentage excess in mortality (%)	
	Counterfactual scenario of grid-specific minimum TV	Counterfactual scenario of no TV variation
Global	2.2 (1.5 to 2.9)	3.4 (2.2 to 4.6)
Americas	1.6 (0.9 to 2.4)	2.7 (1.5 to 4.0)
Northern America	1.9 (1.1 to 2.7)	3.2 (1.8 to 4.5)
Latin America and the Caribbean	1.4 (0.7 to 2.0)	2.3 (1.1 to 3.5)
Europe	1.2 (0.6 to 1.8)	1.6 (0.8 to 2.3)
Northern Europe	1.0 (0.6 to 1.5)	1.5 (0.9 to 2.1)
Eastern Europe	1.1 (0.4 to 1.8)	1.3 (0.5 to 2.2)
Western Europe	1.2 (0.8 to 1.7)	1.7 (1.0 to 2.4)
Southern Europe	1.4 (1.0 to 1.9)	2.1 (1.4 to 2.8)
Africa	1.0 (0.2 to 1.7)	1.7 (0.5 to 3.0)
Northern Africa	1.7 (1.1 to 2.3)	3.6 (2.4 to 4.9)
Sub-Saharan Africa	0.9 (0.1 to 1.6)	1.5 (0.3 to 2.7)
Asia	3.0 (2.3 to 3.8)	4.7 (3.5 to 5.9)
South-eastern Asia	1.7 (1.1 to 2.3)	3.2 (2.1 to 4.3)
Western Asia	3.6 (3.0 to 4.3)	5.7 (4.6 to 6.7)
Central Asia	3.9 (2.9 to 4.9)	5.1 (3.8 to 6.5)
Southern Asia	3.5 (2.6 to 4.4)	5.3 (4.0 to 6.7)
Eastern Asia	2.8 (2.1 to 3.6)	4.3 (3.2 to 5.5)
Oceania	2.1 (0.7 to 3.6)	3.2 (1.0 to 5.4)
Australia and New Zealand	2.5 (1.0 to 4.1)	3.8 (1.5 to 6.1)
Other regions in Oceania	0.8 (-0.3 to 2.0)	1.3 (-0.6 to 3.3)

References:

1. Xu R, Zhao Q, Coelho MSZS, et al. Socioeconomic inequality in vulnerability to all-cause and cause-specific hospitalisation associated with temperature variability: a time-series study in 1814 Brazilian cities. *The Lancet Planetary Health* 2020; **4**(12): e566-e76.
2. Yang J, Zhou M, Li M, et al. Vulnerability to the impact of temperature variability on mortality in 31 major Chinese cities. 2018; **239**: 631-7.
3. Ma C, Yang J, Nakayama SF, Honda YJEi. The association between temperature variability and cause-specific mortality: Evidence from 47 Japanese prefectures during 1972–2015. 2019; **127**: 125-33.
4. Yi WZ, Zhang XL, Pan RB, et al. Quantifying the impacts of temperature variability on hospitalizations for schizophrenia: A time series analysis in Hefei, China. *Science of the Total Environment* 2019; **696**: 133927.
5. Guo Y, Gasparrini A, Armstrong BG, et al. Temperature variability and mortality: a multi-country study. *Environmental health perspectives* 2016; **124**(10): 1554-9.
6. Gasparrini A, Guo Y, Hashizume M, et al. Mortality risk attributable to high and low ambient temperature: a multicountry observational study. *Lancet* 2015; **386**(9991): 369-75.
7. Bhaskaran K, Gasparrini A, Hajat S, Smeeth L, Armstrong B. Time series regression studies in environmental epidemiology. *Int J Epidemiol* 2013; **42**(4): 1187-95.
